# iDICss robustly predicts melanoma immunotherapy response by synergizing genomic and transcriptomic knowledge via independent component analysis

**DOI:** 10.1002/ctm2.70183

**Published:** 2025-01-08

**Authors:** Jiayue Qiu, Nana Jin, Lixin Cheng, Chen Huang

**Affiliations:** ^1^ Dr. Nesher's Biophysics Laboratory for Innovative Drug Discovery, State Key Laboratory of Quality Research in Chinese Medicine & Faculty of Chinese Medicine Macau University of Science and Technology Taipa Macao SAR China; ^2^ Health Data Science Center, Shenzhen People's Hospital First Affiliated Hospital of Southern University of Science and Technology Shenzhen China

1

Dear Editor,

Here, we present a tool named iDIC‐based scoring system (iDICss) that is useful for predicting immunotherapy response and prognostic outcomes in melanoma patients. The core principle of the tool is that specific driver alterations affecting the immuno‐related gene expression and functions,^1^ may be indicative of high tumour mutation burden, a good predictor used to guide immunotherapy decisions in clinical,^2^ and analysis of such interplays may provide novel strategies to improve prediction of immunotherapy response. This tool thereby builds on an immune driver independent components (iDICs) profile, which innovatively integrates the immunogenic properties into transcriptome using the independent component analysis (ICA), a popular matrix decomposition method. Optimized by comparison of multiple machine‐learning models, an iDICss was established, which exhibits a superior performance of prognostic and immune response prediction compared with other published state‐of‐art biomarkers. Our study provides a novel strategy to improve the prediction of immunotherapy response for melanoma, which could be adaptable in numerous clinical prediction situations.

Melanoma is a highly aggressive skin cancer originating from melanocyte transformation, and its incidence has been increasing globally in recent years.^3^ Immune checkpoint blocking immunotherapy is one of the most advanced treatment strategies and significantly improves the survival outcomes for melanoma sufferers. However, high genetic heterogeneity of melanoma results in immune responses occurring in only a small proportion of patients,^4^, ^5^, ^6^ which motivates us to explore a robust biomarker to predict patients’ immunotherapy response and guide treatment decision. Accumulated studies demonstrate that oncogenic driver mutations shape tumor immune microenvironment (TIME), and cause impediments to immunotherapy.^7^, ^8^, ^9^ Hence, the crosstalk between oncogene driver mutations and TIME‐related gene expression alterations may reflect if a patient will respond to immunotherapy. Herein we started by integrating driver gene mutation and expression information via ICA by which we successfully figured out seven key TIME‐driver iDICs, and then established an iDIC‐based scoring system (iDICss) by a comparative analysis of multiple machine‐learning methods. The main pipeline proceeded as follows: (1) independent component analysis, (2) independent component (IC) selection, (3) TIME‐driver IC profile calculation, and (4) iDICss construction (Figure 1). The datasets involved in the study were summarized in Table S1.

**FIGURE 1:**
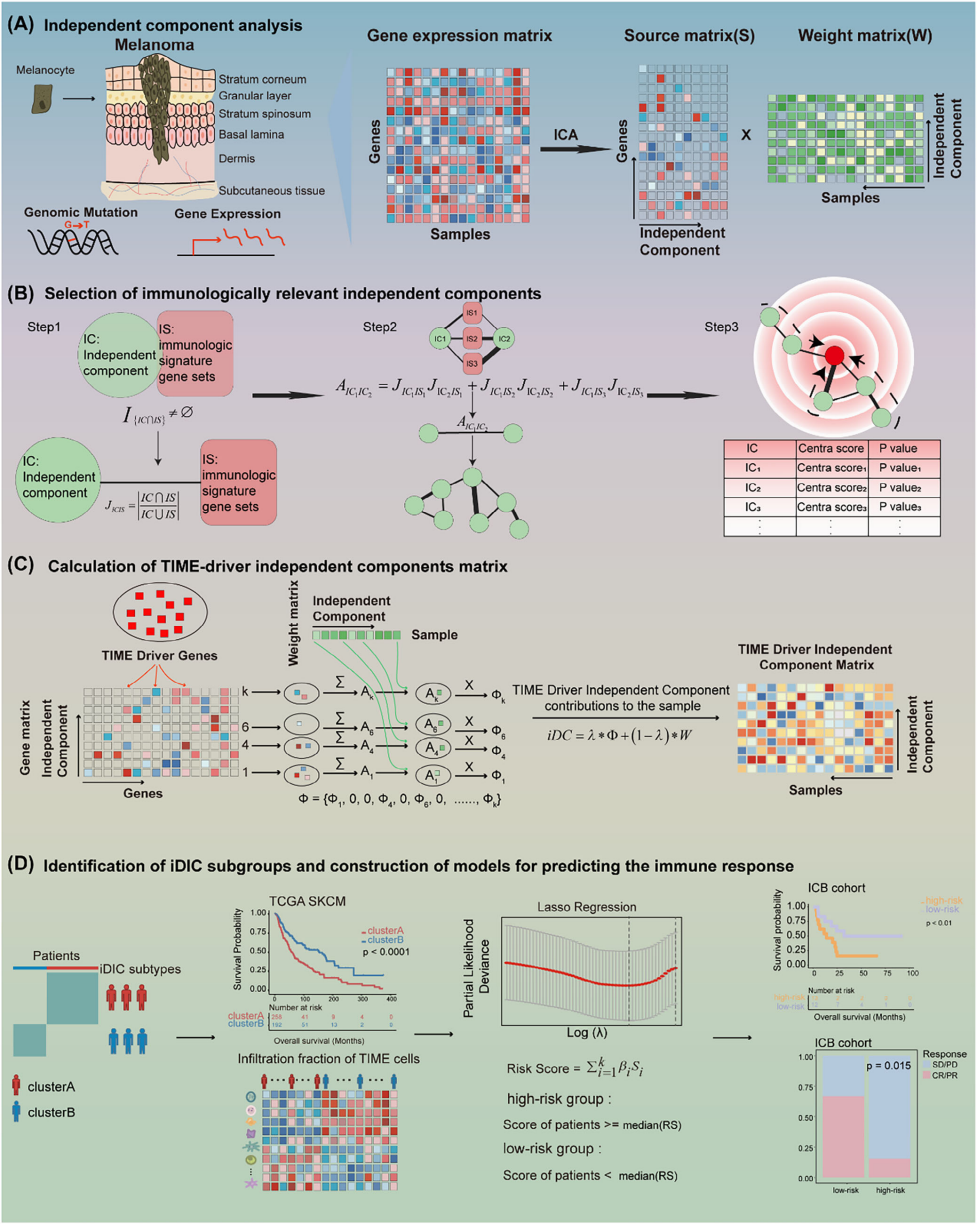
Flowchart for constructing models based on TIME‐driver independent components in melanoma cancer patients. (A) Independent component analysis in TCGA SKCM cohort. (B) Selection of immunologically relevant independent components based on a network method. (C) Calculation of TIME‐driver independent components profile. (D) Identification of iDIC subgroups and construction of a risk model for predicting the immune response.

Briefly, we collected the multi‐omics data of 450 melanoma patients from the TCGA database, including gene expression, mutation as well as clinical information. ICA analysis was initially applied to the gene expression matrix *E*, resulting in an *S* matrix of source‐independent components and a *W* matrix of weight coefficients of each independent component in the sample. We performed independent component crosstalk network analysis to identify ten key ICs significantly associated with the TIME in melanoma (Figure S1, Table S2). The robustness of the key ICs was verified by comparison of the PPI network with a random subnetwork and by mutual information analysis (Figures S2–S4, Table S3). Functional enrichment suggested that these ICs are associated with key immuno‐related pathways, such as T/B cell receptor signaling and inflammatory responses, highlighting their importance in understanding TIME interactions (Table S4). Correlation analysis of key ICs with driver mutations revealed that IC14 was linked to TP53, DCC, and RB1 mutations, while IC99 was linked to BRAF and NRAS mutations. These findings underscore the potential of these ICs to elucidate the interaction between immune responses and genetic alterations in melanoma patients (Figure S5, Table S5). Next, we developed a computational framework to quantify the degree of drive gene mutation impacting those ICs (see Supporting Information Methods). In this step, we introduced a new parameter *λ*, which could be used to balance the weight of drive gene mutation and immunological mutation impacting ICs. In this study, *λ* was set at .7 since it showed the best performance compared with .5, .8, and 1, respectively (Figures S6, S7). Unsupervised clustering of iDIC profiles divided melanoma patients into two subgroups (clusterA and clusterB). ClusterB exhibits a better survival outcome (Figures S6, S7) and is characterized by a higher immune cell infiltration, suggesting a more active TIME (Figure S8, Table S6). Finally, a risk model named iDICss based on 15 signature genes selected by LASSO regression (Figure S9, Table S7) was established, demonstrating prognostic and predictive efficacy superior to the 117 machine‐learning combinatorial models. Patients were stratified into groups of higher and lower risk based on median iDICss scores. In the TCGA SKCM cohort, Kaplan–Meier analyses revealed that patients with lower risk scores experienced markedly prolonged overall survival (OS), with the log‐rank test yielding a *p*‐value less than .001. Additionally, time‐dependent ROC evaluations affirmed the robust prognostic significance of iDICss as an independent variable (Figures S10–S11).

Next, three independent immunotherapy cohorts including GSE115821, GSE100797, and the Gide cohort were used to explore its predictive capability for immunotherapy outcomes. The low‐risk group patients harbour a significantly longer OS and longer progression‐free survival (Figure 2A,B), and ROC analyses validated the predictive value of iDICss (Figure 2D). A higher ORR was also found in the low‐risk group (Figure 2C, Figure S12), indicating potential benefits from immunotherapy for this group. Moreover, we screened out several potential agents that might improve immune response for the low‐risk group, that is, colchicine and HDAC inhibitors (Figure 2F–I).

**FIGURE 2:**
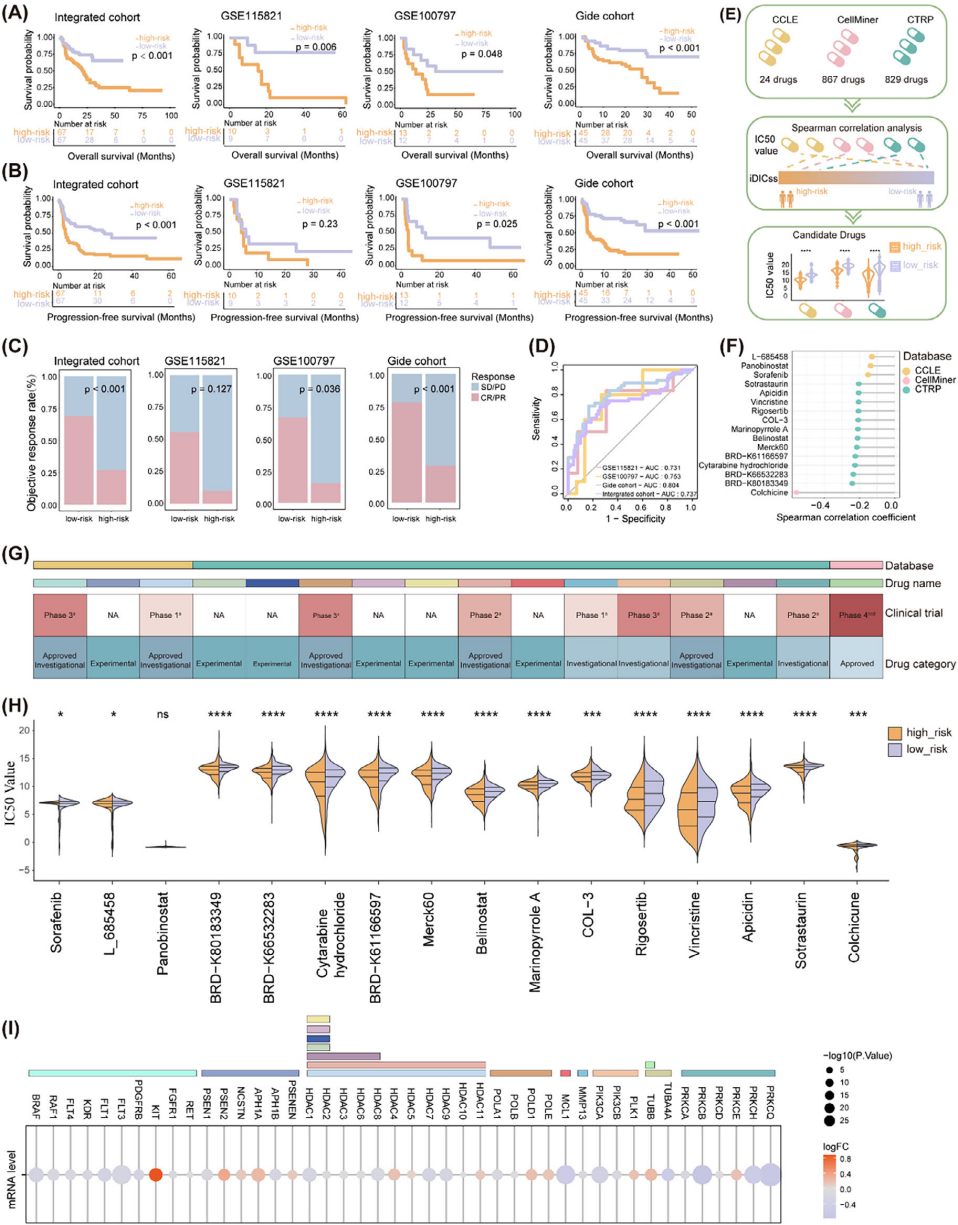
Comparisons of progression‐free survival (PFS) (or OS), ORR, and drug sensitivity between low‐risk and high‐risk groups across multiple cohorts. (A–C) Comparison of OS (A), PFS (B), and ORR (C) between the low‐risk and high‐risk groups in the GSE115821, GSE100797, Gide cohort, and integrated cohorts. The *p*‐values were calculated with the log‐rank test and the Wilcoxon rank sum test, respectively. (D) ROC curve of iDICss for predicting the immune response of patients from the GSE115821, GSE100797, Gide cohort, and integrated cohorts. (E) The procedure for screening drugs by iDICss. (F) Spearman correlations between iDICss and IC50 value of drugs from CCLE dataset. (G) The information of candidate drugs about clinical trial and category (1: melanoma; 2: gout; 3: acute coronary syndrome (ACS); 4: coronavirus disease 2019 (COVID‐19)). (H) Comparison of drug sensitivity (IC50 values) of candidate drugs from the CCLE, CTRP, and CellMiner databases between high‐risk and low‐risk groups. The *p*‐value was estimated using the Wilcoxon rank sum test. ns, *p* > .05, *p < .05; **p < .01; ***p < .001; ****p < .0001; *****p < .00001. (I) Comparison of mRNA level of drug targets between high‐risk and low‐risk groups.

Compared with 117 machine learning models and various established biomarkers, respectively, iDICss exhibits superior performance in predicting prognosis (average C‐index: .725) and immunotherapy outcomes (average C‐index: .76) across different cohorts (Figure 3A–D, Figure S13). Notably, iDICss also outperformed established markers in the five additional independent cohorts (Figure 4A–C). The ROC curves show that iDICss predicts the ORR of patients with high accuracy from .7 to .979 (Figure 4B). These findings suggest that iDICss is a promising biomarker for guiding treatment strategies and predicting clinical benefits for melanoma patients.

**FIGURE 3:**
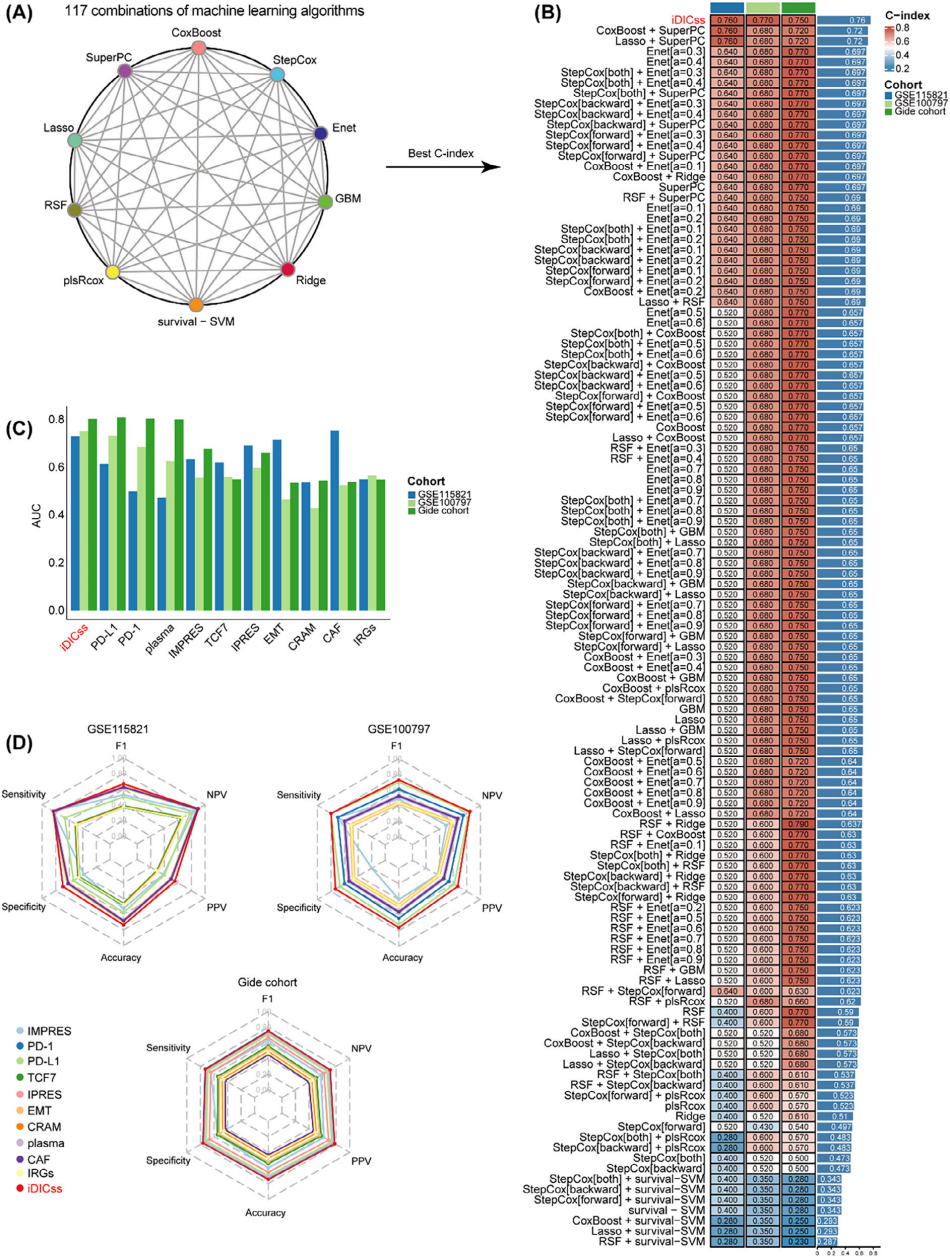
Compared the performance of iDICss with existing models for predicting immune response across multiple cohorts. (A) All combinations of machine learning algorithms (stepwise Cox, CoxBoost, ridge regression, RSF, GBM, Survival‐SVM, LASSO, SuperPC, plsRcox, and Enet). (B) Through a comprehensive computational framework, a combination of 117 machine‐learning algorithms was generated. The C‐index of each model for predicting immune response was calculated through the GSE115821, GSE100797, and Gide cohorts and sorted by the average C‐index. (C) The AUC of iDICss and other published predictors across three available ICB treatment datasets including GSE115821, GSE100797, and Gide cohorts. (D) Performance measurements of IMPRES, PD‐1, PD‐L1, TCF7, IPRES, EMT, CRAM, plasma, CAF, IRGs, and iDICss illustrated by sensitivity, specificity, F1, PPV, NPV, and accuracy.

**FIGURE 4:**
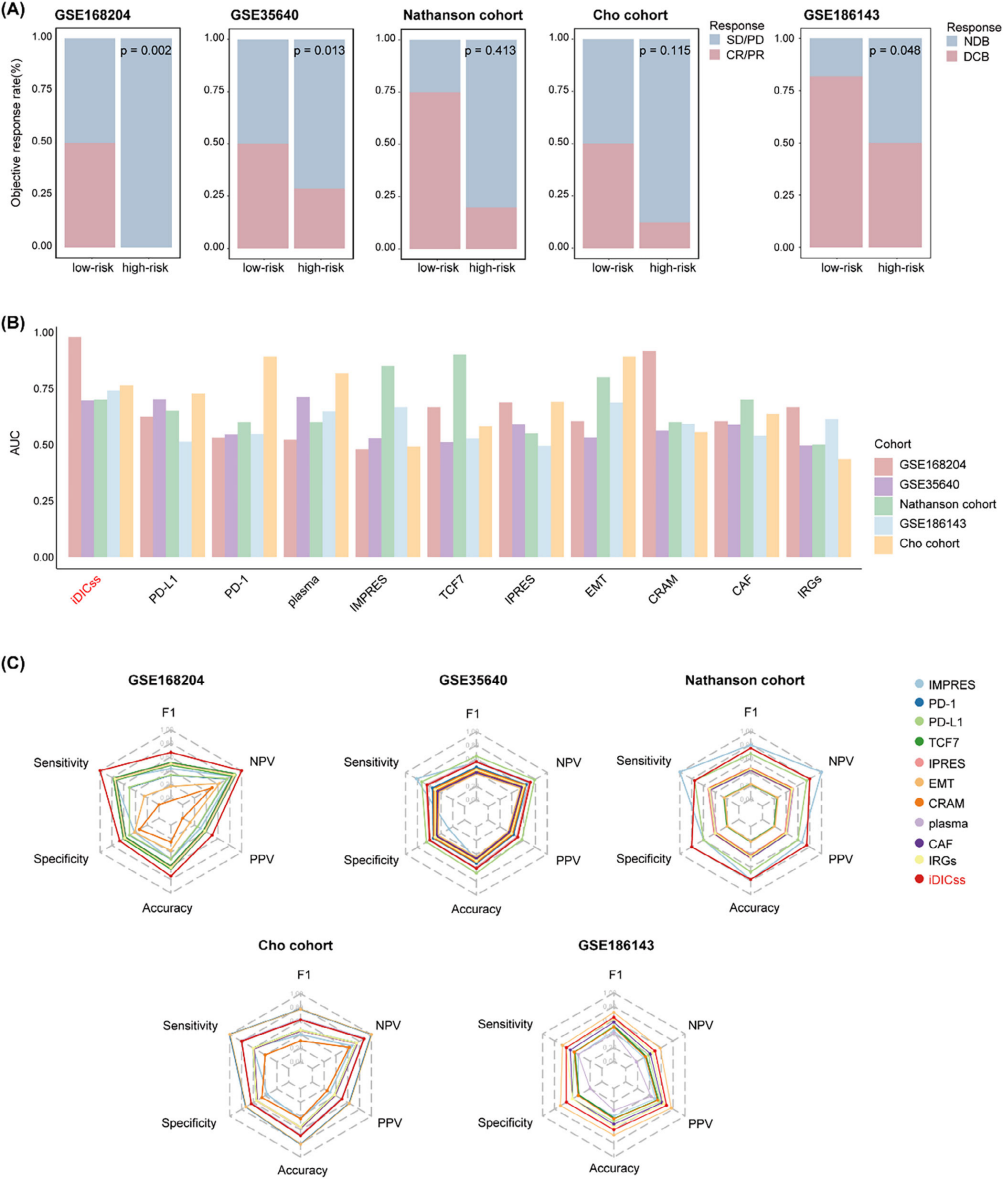
Compared the performance of iDICss with existing models for predicting immune response across multiple cohorts. (A) Comparison of ORR between the low‐risk and high‐risk groups in the GSE168204, GSE35640, Nathanson cohort, Cho cohort, and GSE186143. The *p*‐value was estimated using the Wilcoxon rank sum test. (B) The AUC of iDICss and other published predictors across three available ICB treatment datasets including GSE168204, GSE35640, Nathanson cohort, Cho cohort, and GSE186143. (C) Performance measurements of IMPRES, PD‐1, PD‐L1, TCF7, IPRES, EMT, CRAM, plasma, CAF, IRGs, and iDICss illustrated by sensitivity, specificity, F1, PPV, NPV, and accuracy.

In conclusion, this study proposed a novel approach by which we effectively integrated the traits of TIME driver mutations and TIME gene expression and established a scoring system, demonstrating strong predictive power for the prognostic outcomes and efficacy of immunotherapy in melanoma cases. This approach provides valuable insights into cancer biology and has the potential to enhance clinical decision‐making and optimize immunotherapy strategies for melanoma patients.

## AUTHOR CONTRIBUTIONS

All authors participated in the study planning and analysis or laboratory experiments. Jiayue Qiu: Conceptualization, methodology, validation, formal analysis, investigation, data curation, writing—original draft preparation, writing—review and editing, and visualization. Nana Jin: Formal analysis, investigation, and writing—review and editing. Lixin Cheng: Conceptualization, investigation, writing—review and editing, supervision, project administration and funding acquisition. Chen Huang: Conceptualization, methodology, formal analysis, investigation, resources, writing—review and editing, supervision, project administration, and funding acquisition.

## CONFLICT OF INTEREST STATEMENT

The authors declare no conflict of interest.

## CODE AVAILABILITY

Source code is available at https://github.com/ChenHuangMUST/iDICss.

## Supporting information



Supporting information

## Data Availability

The TCGA SKCM cohort was downloaded from the GDC TCGA data portal (https://portal.gdc.cancer.gov/). Eight external validation cohorts, GSE115821, GSE100797, the Gide et al. cohort, GSE168204, GSE35640, GSE186143, the Cho et al. cohort and the Nathanson et al. cohort were downloaded from the GEO database (https://www.ncbi.nlm.nih.gov/geo/) and BioProject database (https://www.ncbi.nlm.nih.gov/bioproject). The expression data of cell lines and corresponding drug IC50 values from the Cancer Cell Line Encyclopedia (CCLE, https://sites.broadinstitute.org/ccle/), CellMiner (https://discover.nci.nih.gov/cellminer/home.do), and Cancer Therapeutics Response Portal (CTRP, https://portals.broadinstitute.org/ctrp/) databases.
