# Supplementary material for: iDICss robustly predicts melanoma immunotherapy response by synergizing genomic and transcriptomic knowledge via independent component analysis
Source: Clin Transl Med. 2025 Jan 8;15(1):e70183. doi: 10.1002/ctm2.70183 (PMC11707425; doi:10.1002/ctm2.70183)
Supplement: Supplementary file 1 — Supporting information [file CTM2-15-e70183-s001.docx]

**Supplementary Materials for**

**iDICss robustly predicts melanoma immunotherapy response by synergizing genomic and transcriptomic knowledge via independent component analysis**

Jiayue Qiu ^a^, Nana Jin ^b^, Lixin Cheng ^b,^ *, Chen Huang ^a,^ *

**^a^** Dr. Nesher’s Biophysics Laboratory for Innovative Drug Discovery, State Key Laboratory of Quality Research in Chinese Medicine & Faculty of Chinese Medicine, Macau University of Science and Technology, Taipa, Macao SAR, 999078, China

**^b^** Shenzhen People’s Hospital, First Affiliated Hospital of Southern University of Science and Technology, Shenzhen 518020, China

* Corresponding author. Chen Huang, Dr. Neher’s Biophysics Laboratory for Innovative Drug Discovery, State Key Laboratory of Quality Research in Chinese Medicine & Faculty of Chinese Medicine, Macau University of Science and Technology, Taipa, Macao SAR 999078, China. Tel.: +853-6806-9981; Fax: +853-2888-0022; E-mail: chuang@must.edu.mo; or Lixin Cheng, Shenzhen People’s Hospital, The First Affiliated Hospital of Southern University of Science and Technology, Shenzhen 518000, China. Tel.: (86)0755-25533018; Fax: 0755-25533497; E-mail: easonlcheng@gmail.com

**This supplementary materials contents:**

**Figure S1-S13**

**Table S1-S8**

**Supplementary Methods**

**
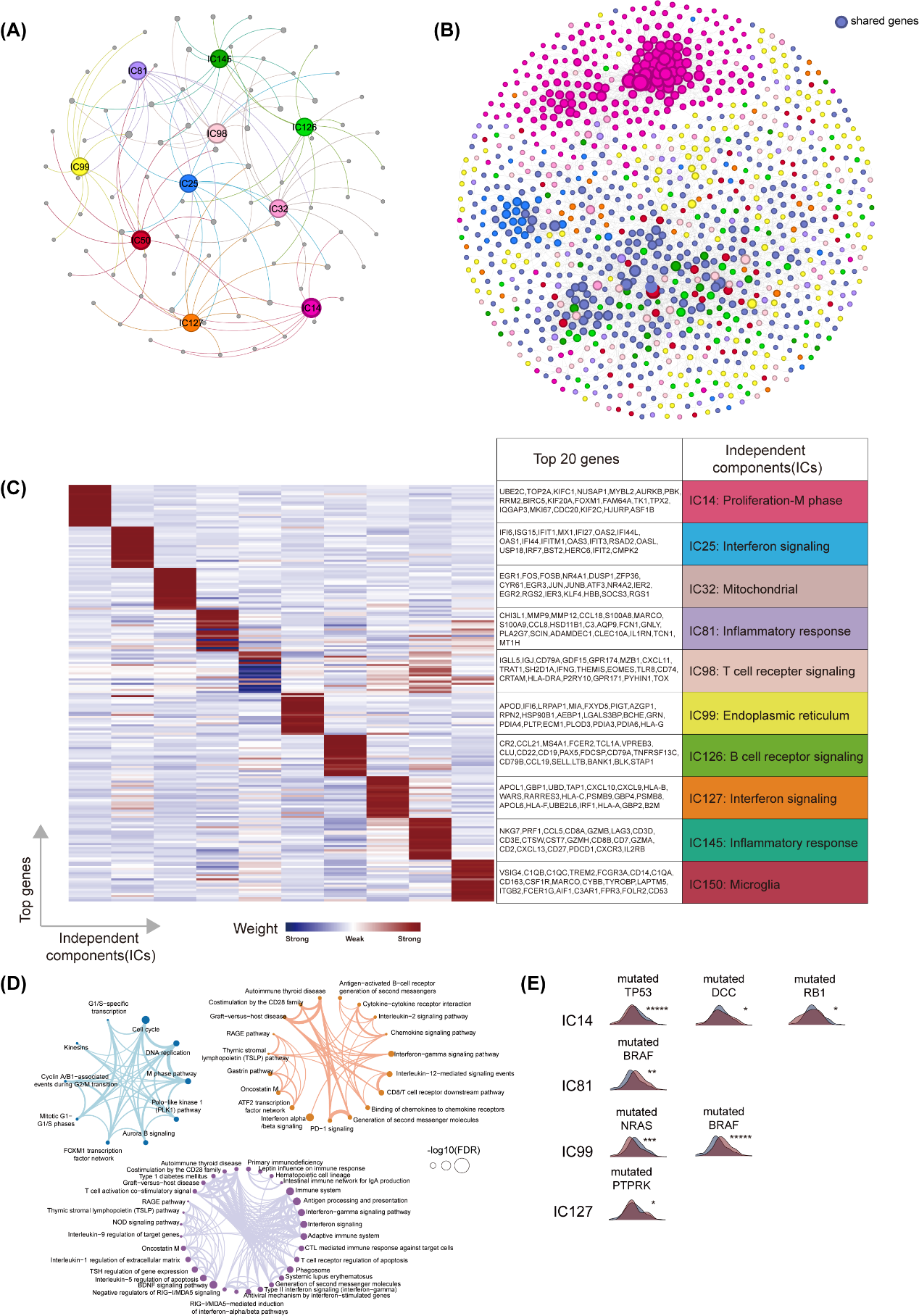
Figure S1. Interpretation of the Independent Components.** **(A)** The correlation between ICs and ISs. (gray node represents IS). **(B)** The PPI network illustrated the interaction of key genes in each immune-related IC. The purple nodes represent key genes shared by two or more ICs, and other nodes are colored consistent with the figure (A). **(C)** Heatmap of the top ten ICs showing normalized gene weights. **(D)** Functional enrichment for the key genes of ICs. **(E)** Density plots showing the associations between component expression and mutations. The X axis represents component expression level, and the Y axis represents patient distribution. Red and blue respectively indicate the distribution of patients positive or negative for mutation as noted in TCGA SKCM cohort. *, FDR < 0.05; **, FDR < 0.01; ***, FDR < 0.001; ****, FDR < 0.0001.

**
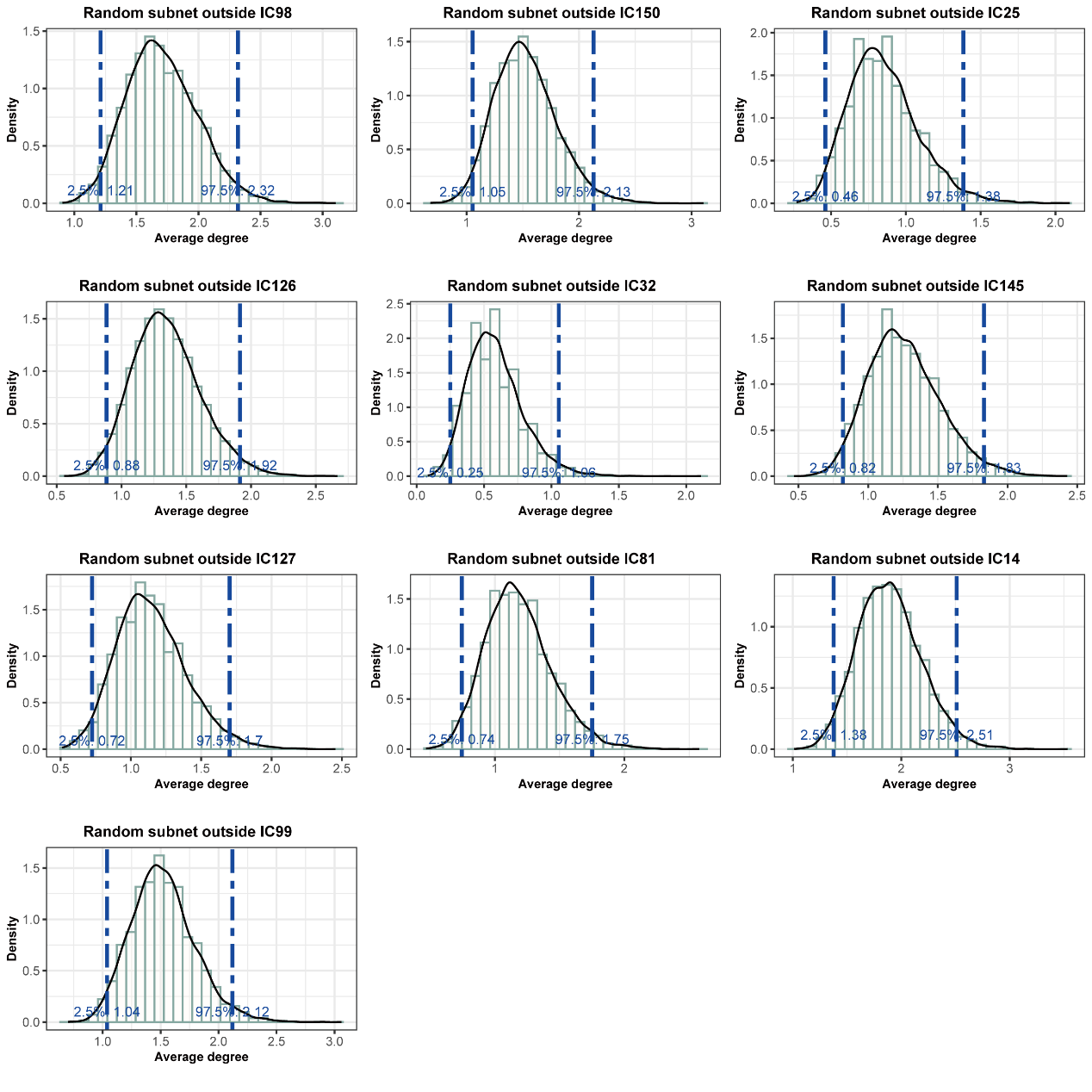
**

**Figure S2. Distribution of the average degree of randomized subnetworks with the same number of nodes for the key genes of ICs.** (The number of components in each iteration was set as the number of components with a 99% variance of the reconstruction calculated from the principal component analysis.)

**
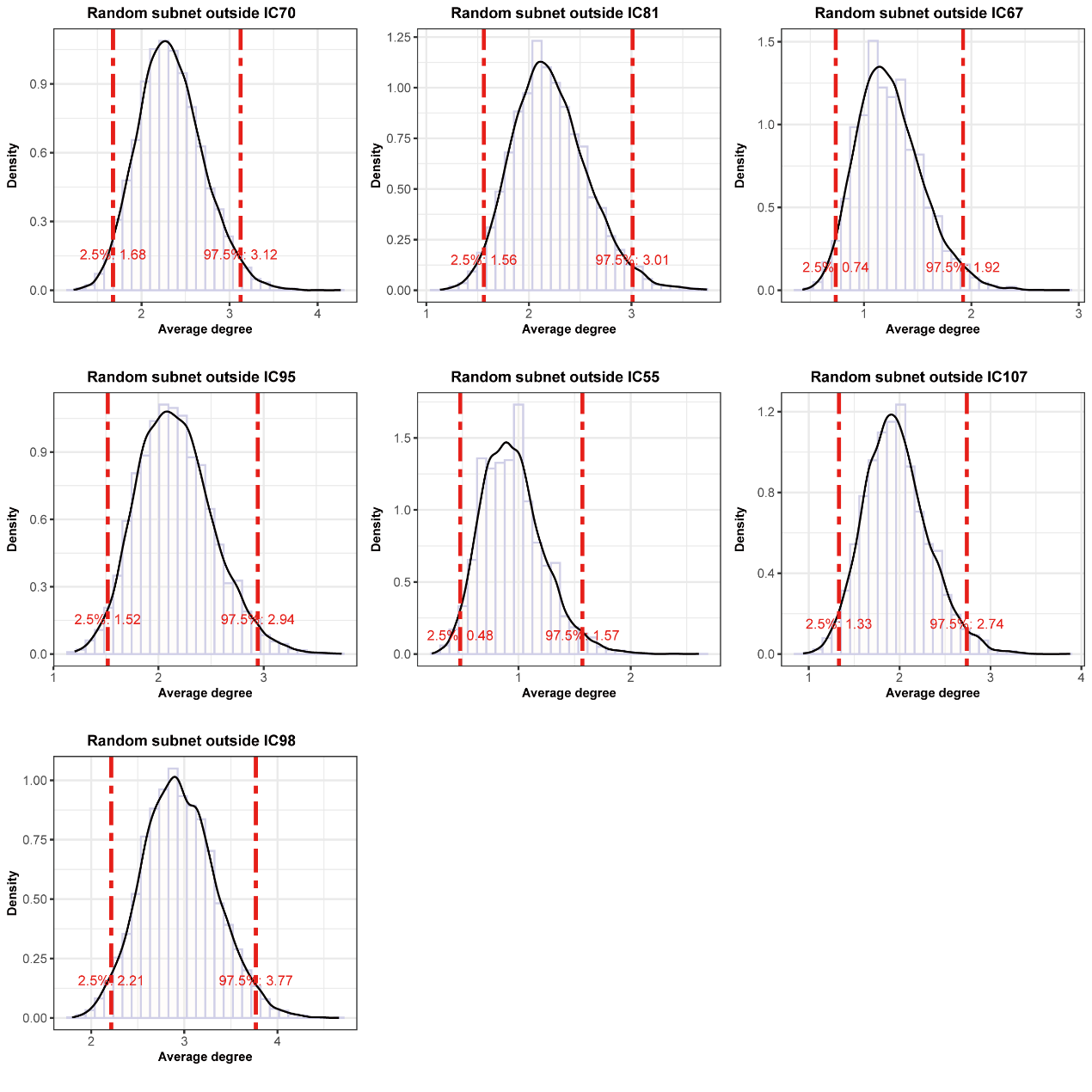
**

**Figure S3. Distribution of the average degree of randomized subnetworks with the same number of nodes for the key genes of ICs.** (The number of components in each iteration was set as the number of components with a 90% variance of the reconstruction calculated from the principal component analysis.)

**
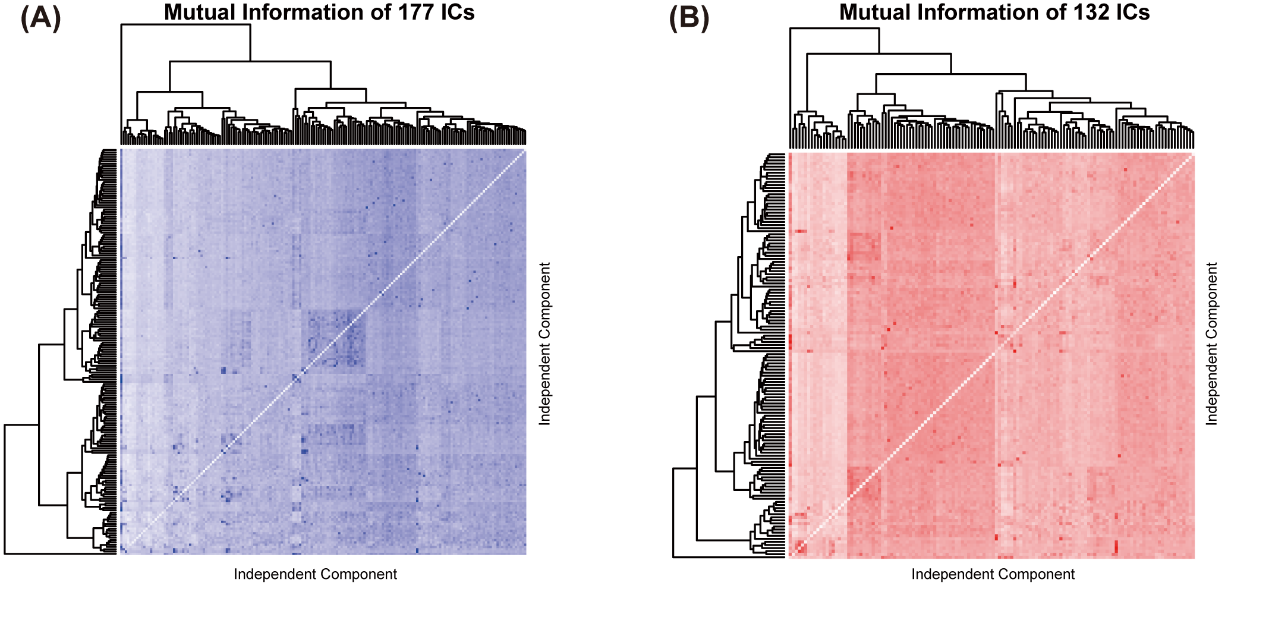
**

**Figure S4. The Mutual information matrix of ICs. (A)** The 177 ICs obtained by the ICA analysis with the number of components with a 99% variance of the reconstruction calculated. **(B)** The 132 ICs obtained by the ICA analysis with the number of components with a 90% variance of the reconstruction calculated.

**
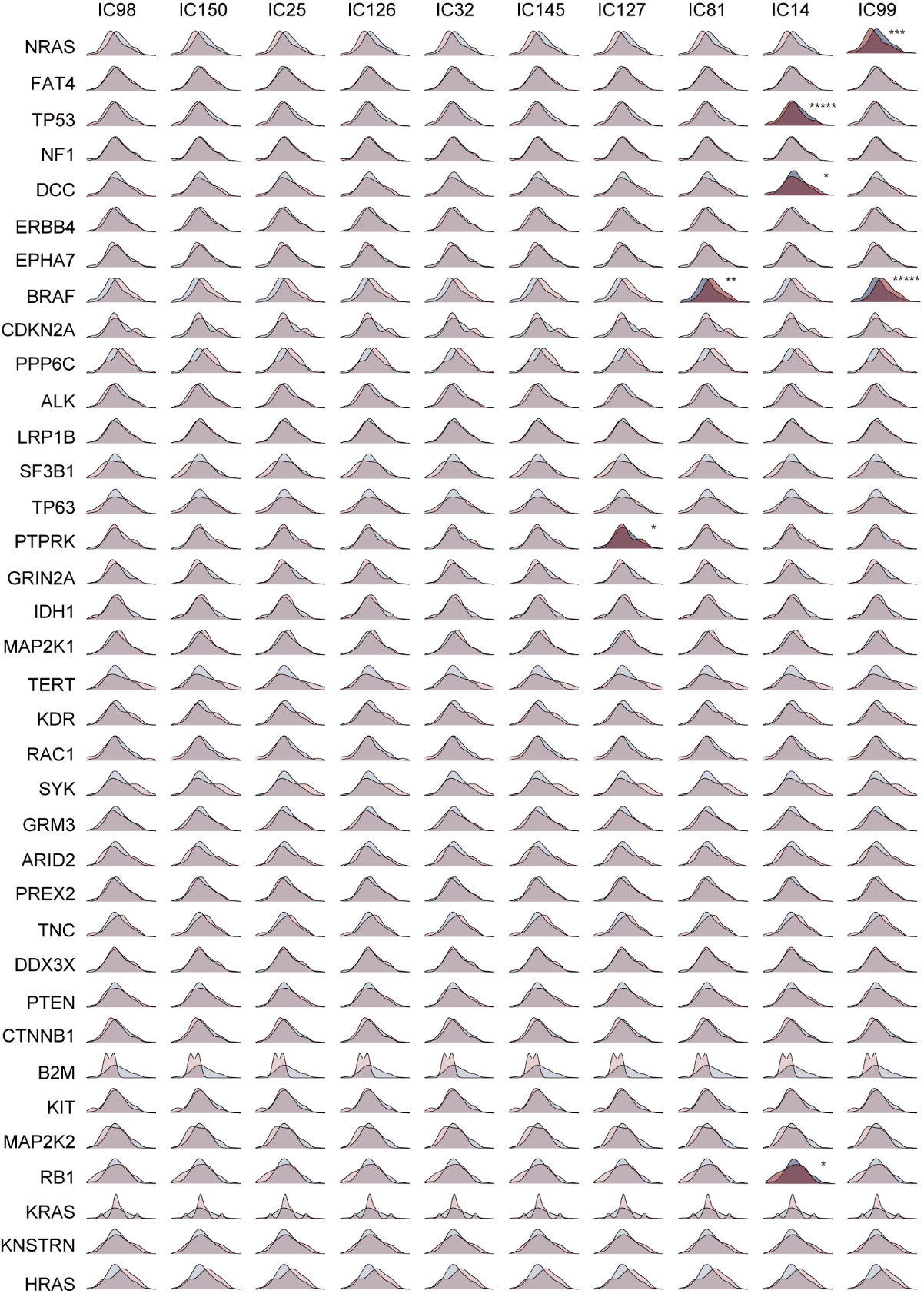
**

**Figure S5. Selected ICs expression associations with driver genes mutations of melanoma.** ∗∗, FDR < 0.01; ∗∗∗, FDR < 0.001; ∗∗∗∗, FDR < 0.0001; ∗∗∗∗∗, FDR < 0.00001.


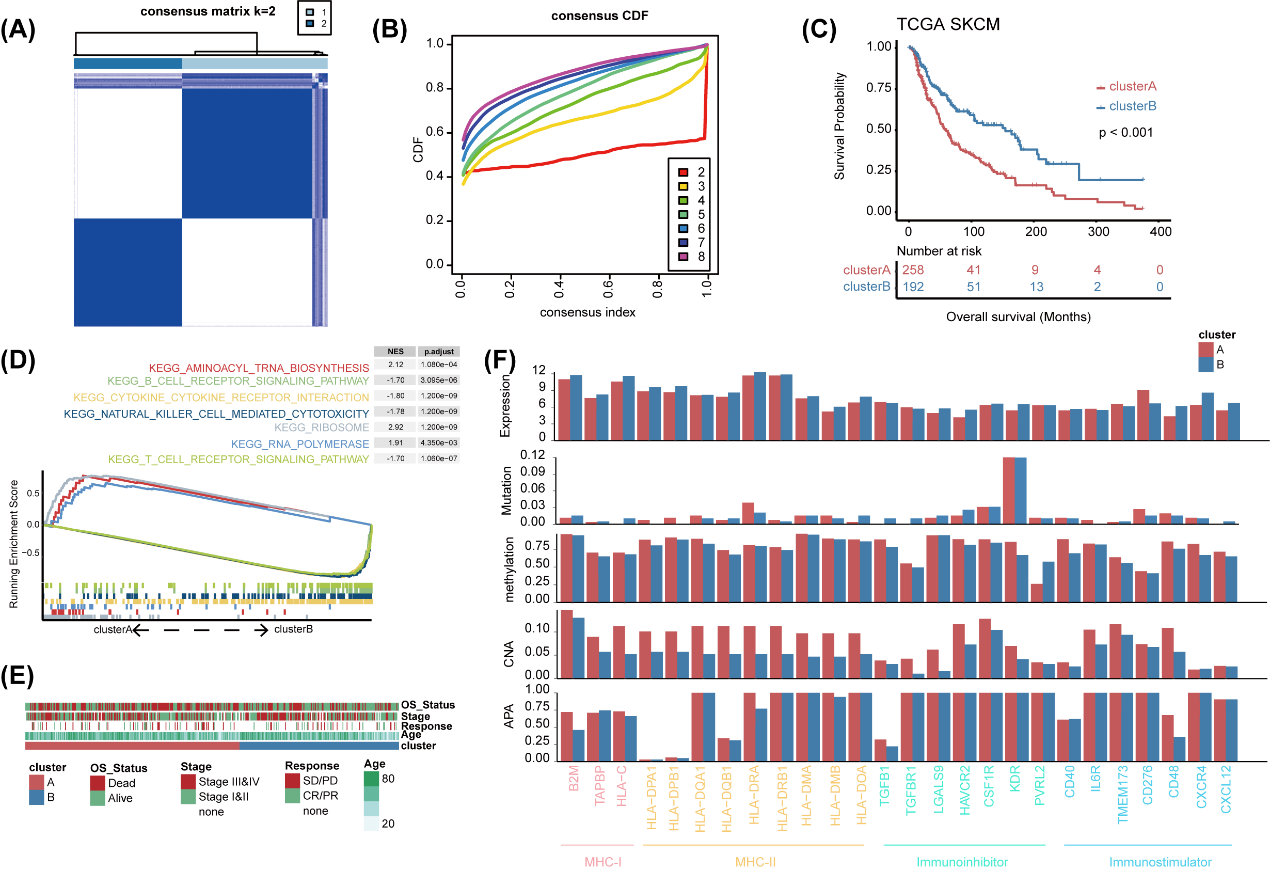


**Figure S6.** Identification of iDIC subgroups. **(A)** The consensus score matrix of all samples when k = 2. A higher consensus score between two samples indicates they are more likely to be grouped into the same cluster in different iterations. **(B)** The CDF curves of the consensus matrix for each k (indicated by color). **(C)** Kaplan-Meier curves for overall survival (OS) of all melanoma patients with two iDIC subgroups. The log-rank test showed an overall p < 0.001. **(D)** GSEA plot of significant KEGG pathways in comparison between the clusterA and clusterB groups. **(E)** Distribution of iDIC subgroups, age, response, stage and os_status in the TCGA SKCM cohort. **(F)** Gene-level summary of multi-omics features in the TCGA SKCM samples. Bar plots depicted the expression, somatic mutations, DNA copy number variations, DNA methylation and APA of a list of immune genes.

**
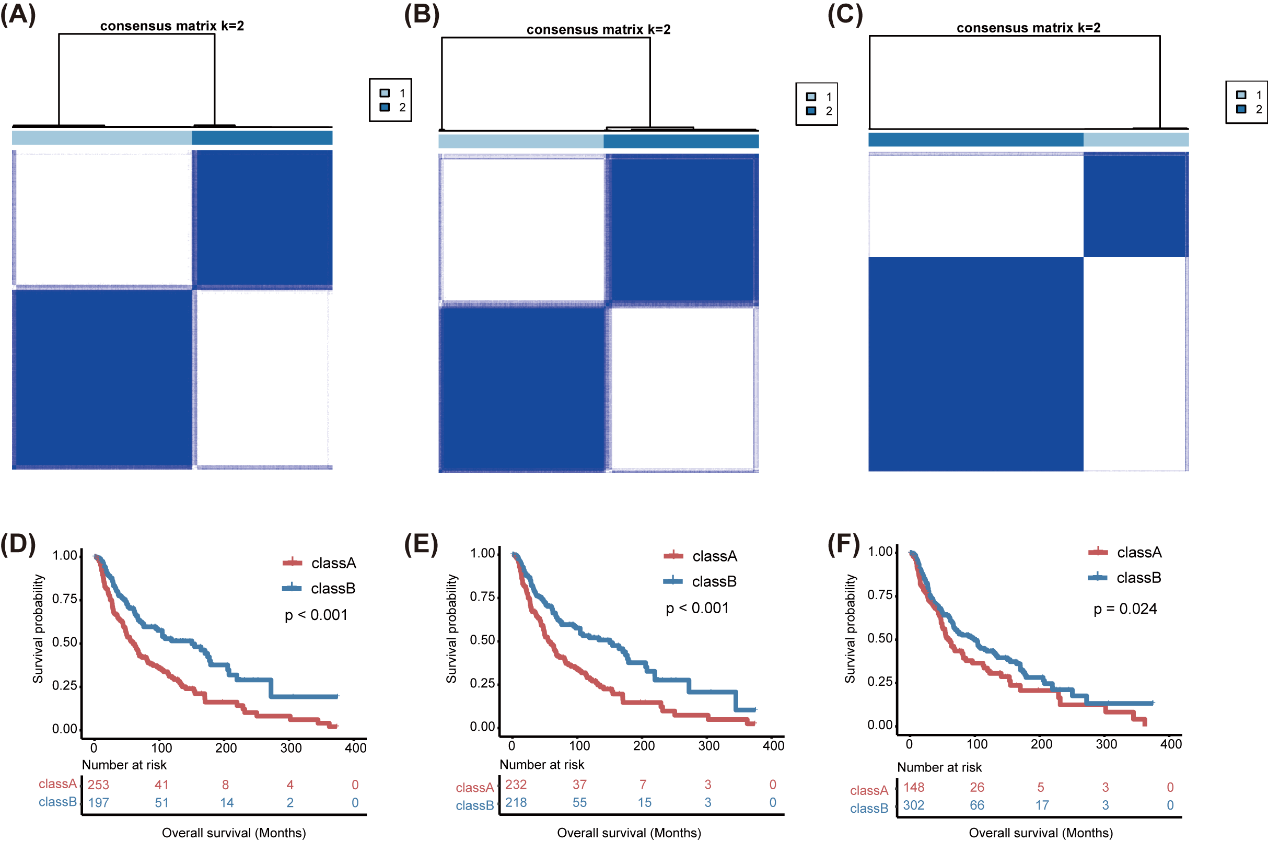
**

**Figure S7. Consensus matrix of all SKCM samples when λ is 0.5, 0.8, and 1, respectively, displaying the stability of clustering after 1000 iterations of hierarchical clustering. (A)-(C)** The consensus score matrix of all samples when λ set to 0.5 (A), 0.8 (B), and 1 (C). **(D)-(F)** Kaplan-Meier curves for overall survival (OS) of all melanoma patients in two clusters when λ set to 0.5 (D), 0.8 (E), and 1 (F). The P-values were calculated with the log-rank test.


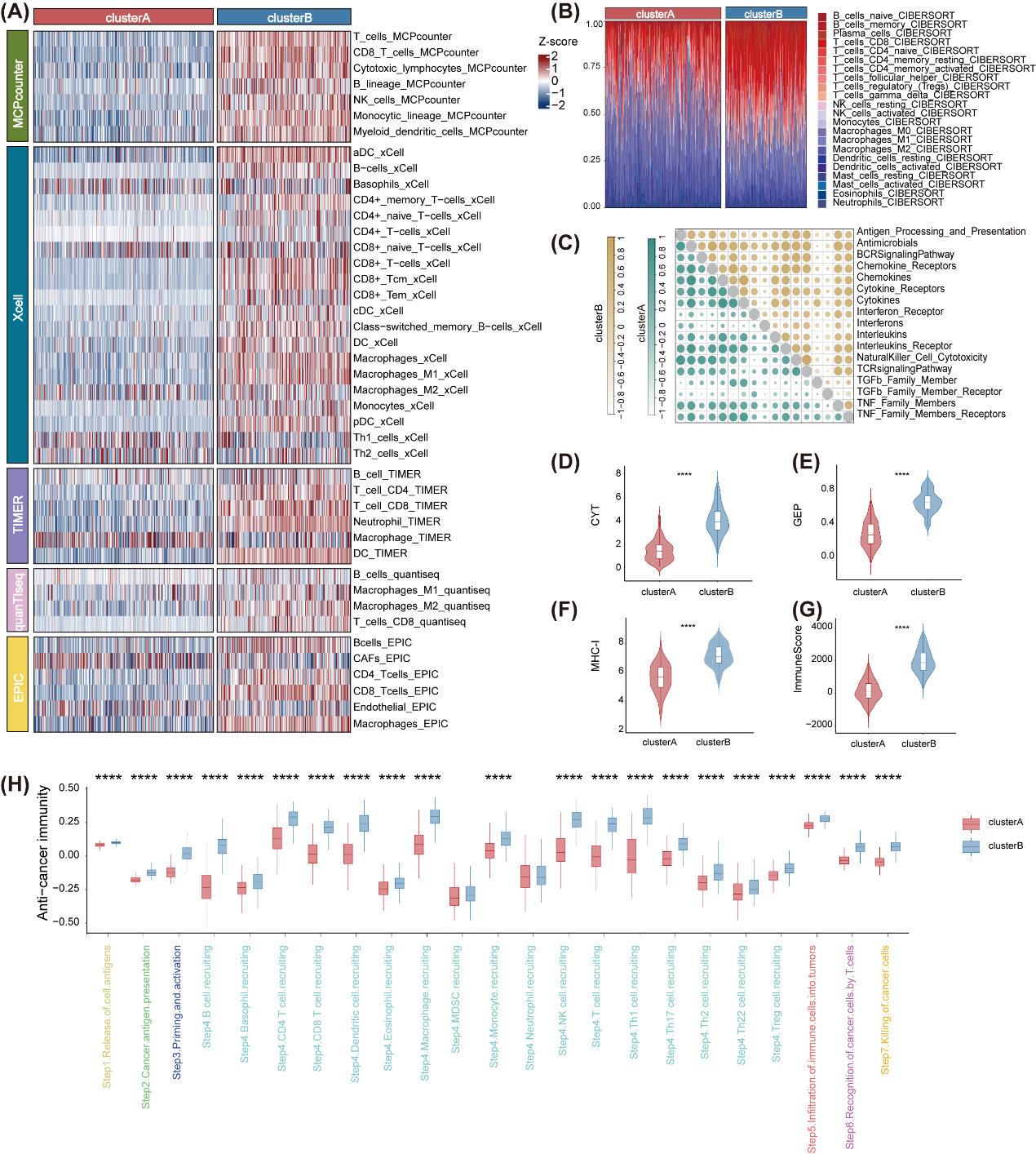


**Figure S8.** The different infiltration patterns between iDIC subgroups. **(A)** The infiltration abundance of immune cells was evaluated by MCP-counter, Xcell, TIMER, quanTIseq and EPIC algorithms for two iDIC subgroups. **(B)** The distribution of immune cell subset infiltration was calculated by the CIBERSORT algorithm. **(C)** Correlations of 17 immune signatures in the two iDIC subgroups. **(D)-(G)** Comparison of the CYT score (D), GEP score (E), MHC-I score (F), and immune score (G) between the clusterA and clusterB groups. **(H**) Differences in the various steps of the cancer immunity cycle between iDIC subgroups. *, p < 0.05; **, p < 0.01; ***, p < 0.001; ****, p < 0.0001.

**
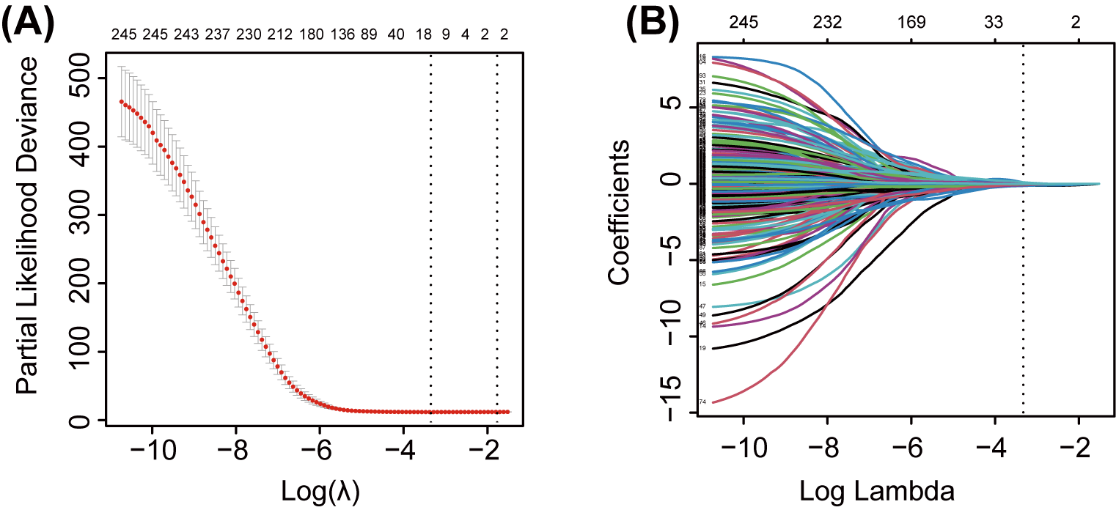
**

**Figure S9. (A)** LASSO regression analysis showed the partial likelihood deviation curve of the minimum number genes corresponding to the iDIC subgroups. **(B)** LASSO coefficient profiles of the fifteen potential genes.

**
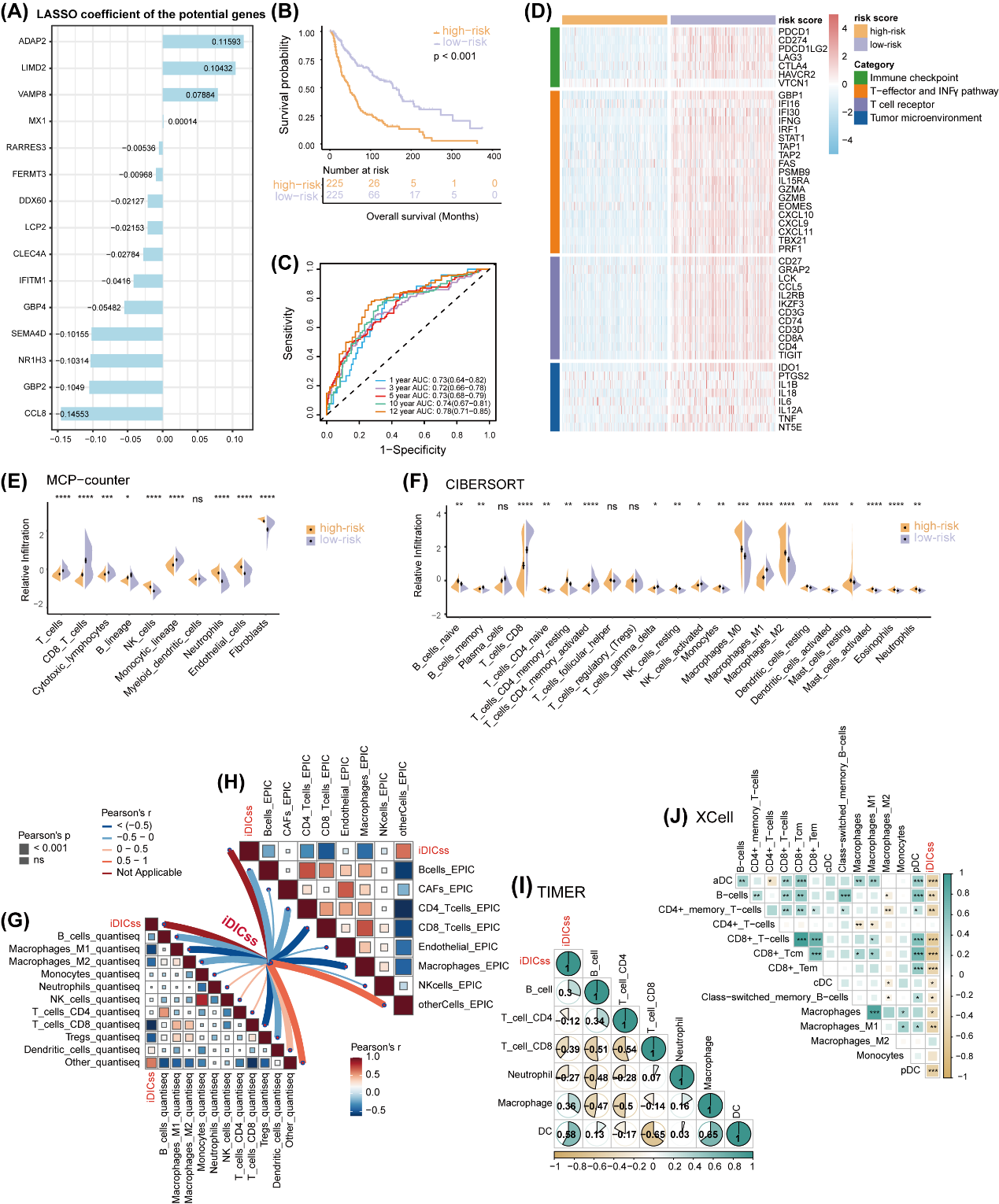
**

**Figure S10.** Construction of iDIC-based risk score signature. **(A)** LASSO coefficient profiles of the fifteen potential genes. **(B)** Kaplan-Meier survival curves of OS comparing the clusterA and clusterB groups from TCGA SKCM cohort. The P-values were calculated with the log-rank test. **(C)** Time-dependent ROC curves for prognosis of the iDIC-based risk score model for 1-, 3-, 5-, 10- and 12- years OS in the TCGA SKCM cohort. **(D)** Differences in the expression of immune response-related genes including immune checkpoints, T-effector and IFNγ pathway-relevant genes, T-cell receptors, and tumor microenvironment genes, between low-risk and high-risk groups in TCGA SKCM cohort. **(E)-(F)** Comparisons of the distribution of immune cells infiltration using MCP-counter (E), CIBERSORT (F) between patients in high-risk and low-risk groups. **(G)- (J)** Pearson correlations between iDICss and fractions of immune cells using quanTIseq (G), EPIC (H), TIMER (I) and Xcell (J) quantification method. *, p < 0.05; **, p < 0.01; ***, p < 0.001; ****, p < 0.0001; ns, no significance. The P-value was estimated using the Wilcoxon rank sum test.

**
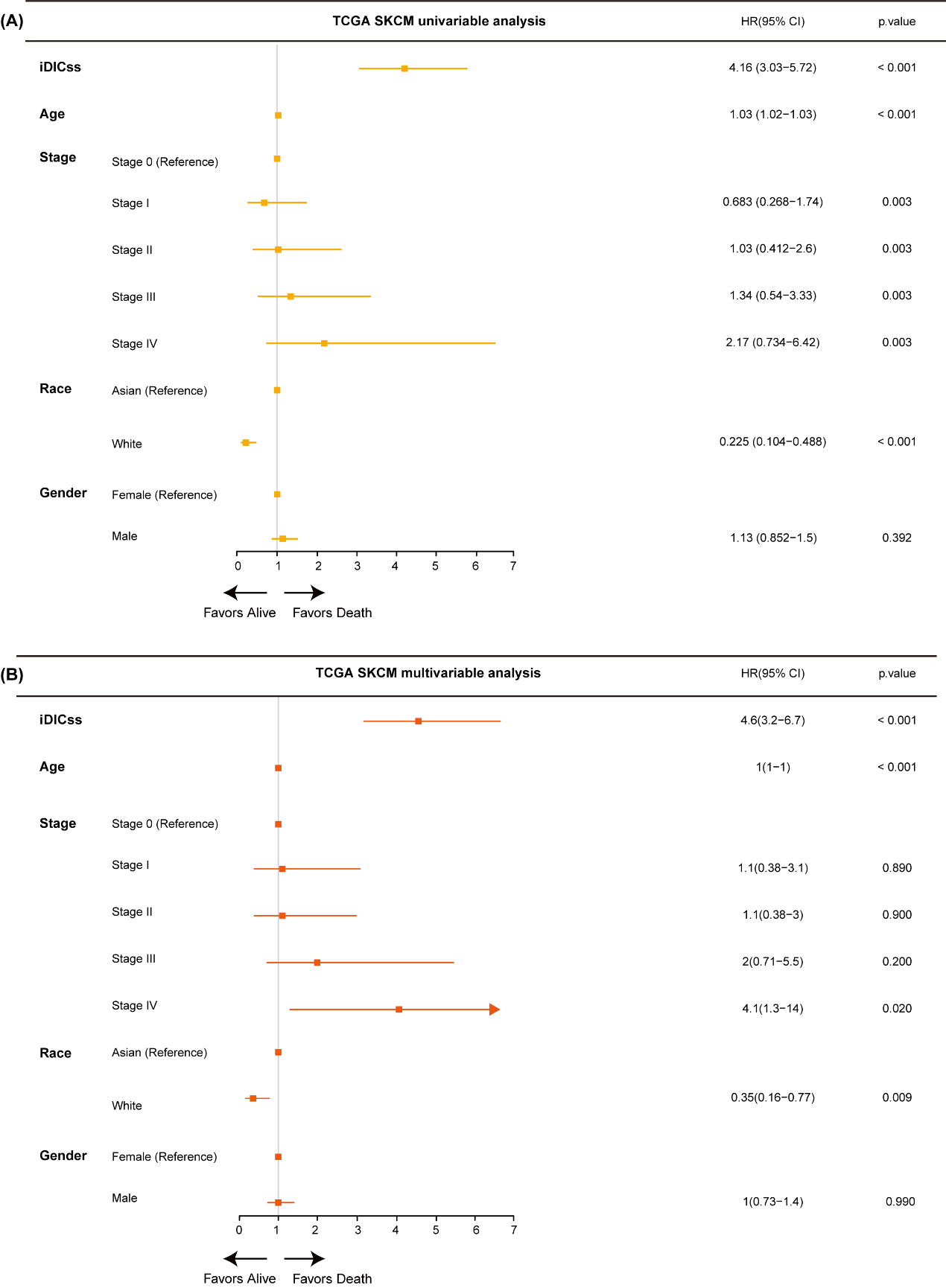
**

**Figure S11.** Univariable and multivariable Cox regression analysis of iDICss and clinicopathological factors (Age, Stage, Race and Gender) for OS in the TCGA SKCM cohort. **(A)** Univariable Cox regression analyses of iDICss and clinicopathological factors (Age, Stage, Race and Gender) for OS in the TCGA SKCM cohort. **(B)** Multivariable Cox regression analyses of iDICss and clinicopathological factors (Age, Stage, Race and Gender) for OS in the TCGA SKCM cohort.

**
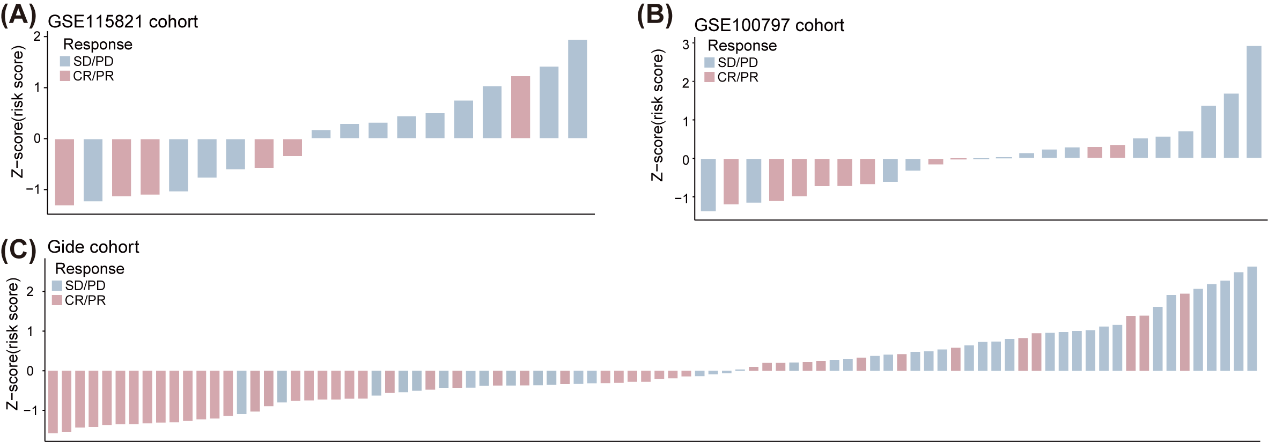
**

**Figure S12. Distribution of the iDICss with different immunotherapy responses.** **(A)-(C)** Comparison of ORR between the low-risk and high-risk groups from the GSE115821 (A), GSE100797 (B) and Gide cohorts (C).

**
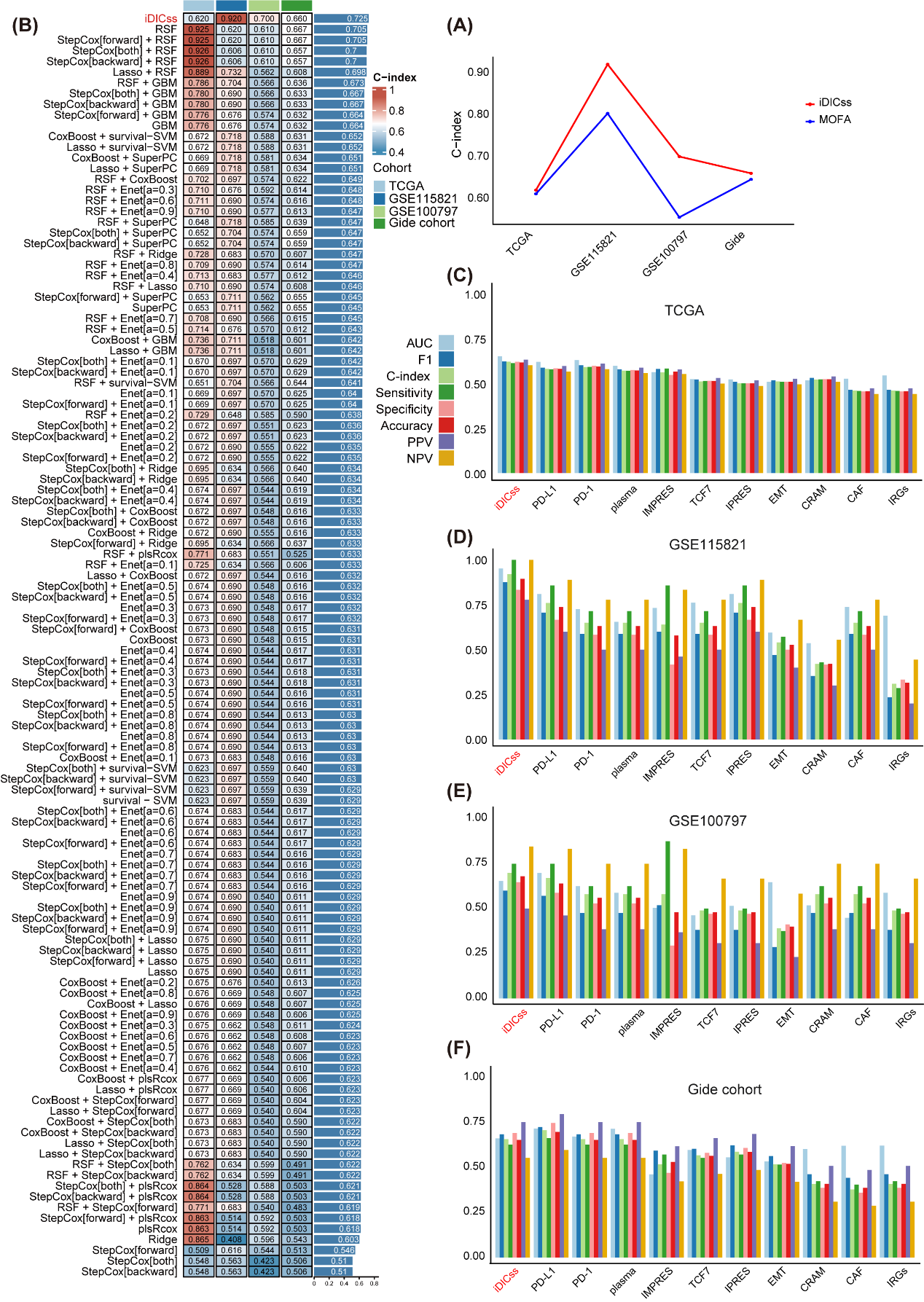
**

**Figure S13.** Compared the performance of iDICss with existing models for predicting prognosis (overall survival) across multiple cohorts. **(A)** Comparison of the C-index for predicting prognosis between iDICss and MOFA in the TCGA, GSE115821, GSE100797 and Gide cohorts. **(B)** Through a comprehensive computational framework, a combination of 117 machine learning algorithms was generated. The C-index of each model for predicting prognosis was calculated through the TCGA, GSE115821, GSE100797 and Gide cohorts and sorted by the average C-index. **(C)-(F)** Performance measurements of iDICss, PD-1, PD-L1, plasma, IMPRES, TCF7, IPRES, EMT, CRAM, CAF and IRGs illustrated by AUC, sensitivity, specificity, F1, PPV, NPV and accuracy in the TCGA (**C**), GSE115821 (**D**), GSE100797 (**E**) and Gide cohorts (**F**).

**Table S1. Data source.**

| **Data source** | **Tumor** | **N** | **RNAseq** | **Clinical outcome** | |
| --- | --- | --- | --- | --- | --- |
| TCGA | Melanoma | 450 | 450 | | OS |
| GSE115821 | Melanoma with  anti-PD-(L)1/ plus  anti-CTLA-4  therapy | 19 | 19 | | ORR  OS  PFS |
| GSE100797 | Melanoma with adoptive T cell therapy | 25 | 25 | | ORR  OS  PFS |
| Gide cohort | Melanoma with  anti-PD-(L)1 therapy | 90 | 90 | | ORR  OS  PFS |
| GSE168204 | Melanoma with  anti-PD-(L)1 therapy | 16 | 16 | | ORR |
| GSE35640 | Melanoma with  anti-MAGE-A3 therapy | 56 | 56 | | ORR |
| Nathanson cohort | Melanoma with  anti-CTLA-4  therapy | 9 | 9 | | ORR |
| Cho cohort | NACLC with  anti-PD-(L)1 therapy | 16 | 16 | | ORR |
| GSE186143 | Melanoma with  anti-PD-(L)1/ plus  anti-CTLA-4  therapy | 27 | 27 | | ORR |

OS, overall survival; PFS, progress-free survival; ORR, objective response rate, the tumor immunotherapy response was defined by the Response Evaluation Criteria in Solid Tumors 1.1 (RECIST 1.1), and patients who experienced a complete response (CR) or partial response (PR) were classified as responders; patients who experienced stable disease (SD) or progressive disease (PD) were classified as non-responders. Especially, for the GSE186143 cohort, patients were classified as a durable clinical benefit (DCB) (complete response [CR]/partial response [PR] or stable disease [SD] that lasted > 6 months) or no durable benefit (NDB) (progression of the disease [PD] or SD that lasted ≤ 6 months).

**Table S2. Independent components are significantly associated with immunologic signature gene sets.**

| **Independent Component (IC)** | **Eigenvector centrality (EC) score** | **P value** |
| --- | --- | --- |
| IC98 | 0.0098 | <0.001 |
| IC150 | 0.0096 | 0.006 |
| IC25 | 0.0094 | 0.010 |
| IC126 | 0.0088 | 0.015 |
| IC32 | 0.0087 | 0.022 |
| IC145 | 0.0087 | 0.026 |
| IC127 | 0.0086 | 0.032 |
| IC81 | 0.0079 | 0.041 |
| IC14 | 0.0079 | 0.045 |
| IC99 | 0.0076 | 0.049 |

**Table S3. The information of the subnetwork in the PPI network**

| **IC** | **N_nodes** | **Degree_IC** | **Degree_random** | **Avg_degree_IC** | **Avg_degree_random [95%CI]** |
| --- | --- | --- | --- | --- | --- |
| Comp98 | 208 | 464 | 355.27 | 2.23 | 1.71[1.21-2.31] |
| Comp150 | 186 | 670 | 283.81 | 3.6 | 1.53[1.05-2.13] |
| Comp25 | 104 | 696 | 88.5 | 6.69 | 0.85[0.46-1.38] |
| Comp126 | 165 | 570 | 222.59 | 3.45 | 1.35[0.88-1.92] |
| Comp32 | 72 | 188 | 42.06 | 2.61 | 0.58[0.25-1.06] |
| Comp145 | 154 | 610 | 194.15 | 3.96 | 1.26[0.82-1.83] |
| Comp127 | 141 | 516 | 161.74 | 3.66 | 1.15[0.72-1.70] |
| Comp81 | 145 | 230 | 171.62 | 1.59 | 1.18[0.74-1.75] |
| Comp14 | 231 | 3342 | 437.24 | 14.47 | 1.89[1.38-2.51] |
| Comp99 | 185 | 482 | 281.34 | 2.61 | 1.52[1.04-2.12] |

**Table S4. Independent component Annotation.**

**Table S5. Driver genes list.**

| **Classification** | **Gene Symbol** |
| --- | --- |
| **Driver genes of melanoma** | ALK, ARID2, B2M, BRAF, CDKN2A, CTNNB1, DCC, DDX3X, EPHA7, ERBB4, FAT4, GRIN2A, GRM3, HRAS, IDH1, KDR, KIT, KNSTRN, KRAS, LRP1B, MAP2K1, MAP2K2, NF1, NRAS, PPP6C, PREX2, PTEN, PTPRK, RAC1, RB1, SF3B1, SMARCB1, SYK, TERT, TNC, TP53, TP63 |
| **Driver genes of TIME** | IL32, FGF23, ESM1, MASP2, CBL, RLN1, IL27RA, XCL1, IFNA1, CD1C, IL12A, CD79B, PPP4C, BTLA, GDF9, CXCR1, MYH7, CFHR2, LYZ, ISG20L2, NR2F1, PTGS2, ZZEF1, NPPB, FGFR4, WAS, PPARA, PDIA3, NRK, CRH, RBM12, QRFP, FCGRT, IGF1R, FGF10, IL10RA, TYK2, NR5A2, NOTCH2, IFNA16, MCL1, FCRL1, NR6A1, KIF1B, FAT3, RXRB, EIF2AK2, INTS4, BMP8A, STAT1, TSC2, POMC, HSPA1L, CDH11, HCFC2, NEK2, PCBP1, ANO4, RAC2, TNFAIP3, ZFHX3, CD96, LARP4B, GPR50, RXRA, SHC4, BMP7, IL9, VIPR1, LRP1, PDK1, ATP8B2, FGF11, IL20, PROC, INPP4A, S100A6, ATP10A, NPR3, EYA4, FGF20, PTH, PIK3R3, FHIT, VCAM1, CUL1, VDR, ACVR1C, PLAG1, MPO, ACVR2B, PTX3, PRKX, EXOC2, TNFRSF17, CNTFR, THPO, NTF4, TYMP, PML, FGF17, CSMD3, TNFRSF14, CD1A, CCL28, MSTN, GABRA6, CYP7B1, HSPA5, TP63, CXCL9, HLA-F, PXDN, NFKBIE, GNAI1, BMP8B, ADRB1, HDAC2, ERBB3, RARB, SOX2, PI4KA, CRHR1, ELN, LEFTY2, TGS1, FLT3LG, GNRHR, MPL, DNAH9, ZAP70, BRD7, FYN, SEMA3A, TK2, BIRC6, HTR3A, GBP2, CCL4L1, TOR2A, CARD6, IL11RA, SETDB1, SDC3, RABEP1, ZYX, PPIA, LGR4, AKAP9, NR2C1, SMAD3, GDF2, NFATC4, HNRNPL, CCL11, RLN3, LTB, APOBEC3C, NRP1, FLT3, UGT2A2, CTCF, ADCYAP1R1, MTNR1B, CCR9, STC1, IL20RA, WNT5B, PPM1L, TUBB3, NMBR, PTPRT, JAK2, TNFRSF13B, IFNG, PVR, GRN, EBI3, GNA11, C3, TRIM22, CLSTN2, FAM3B, THRA, BAZ2B, HTN3, ACTA1, MLLT10, FMR1, MAGEA1, DICER1, PPP3R2, CXCL14, MBD6, CCL8, PLXNC1, NUP133, TNFRSF10C, RETN, SEMA4B, OR10H2, OPRM1, CDKN1A, ANGPTL4, PLXNA4, CDNF, PDYN, IL1RN, CSF3R, HRAS, IL24, LTBP1, BCL9, BMP5, CGB8, STC2, TNFSF4, RLN2, HAMP, LAT, SIRPA, CD74, MTNR1A, NAMPT, SAA1, AHNAK, RHOA, PTPN2, SEMA4G, DOCK8, CD1B, PRLR, FSHR, ACKR4, GPR17, BTNL9, UTS2, CSNK2A1, FXR1, PIK3CA, UBR5, CGA, NR1I2, SAA2, LAG3, FBXO32, RARG, STAB1, TCF7L2, FGF3, ICAM2, SEMA5B, OFD1, RPL22, CBLB, TGFBR1, WDR93, DDX6, AKT2, EP300, CMTM8, SEMA3B, CDK4, FGF19, PGF, RARA, CCL7, IL22RA2, VEGFA, PRRG1, ICOS, HLA-B, IFIH1, MED16, TCF12, INSR, GAST, RNASE2, PPY, CSPG5, IFNK, AHCTF1, NUDT10, IFNW1, PTPN11, SLC6A11, TDRD10, MYC, MAP3K8, LGR6, SPINK5, PLXNA1, FCGR3A, IL17B, NFAT5, GRID1, TDO2, POLE, SMARCB1, IGF1, TRPM1, IL3, CIC, LCP2, MAP2K4, BLNK, NOS2, PRKCQ, SP1, STAG2, IL20RB, MBD1, SH3BP2, SKIV2L, HLA-DRB1, OPRL1, VIP, LRSAM1, ZNF471, PLXND1, FAM126B, PPP6C, HORMAD1, RPRD1B, GH2, OPRD1, HSPA2, IFNA2, CD47, PRL, IL21R, IL4R, ETNK1, PTCH1, DEFB104A, ASB9, CX3CL1, ACKR2, KMT2A, PBRM1, GAB3, HNF4G, HLA-DMA, AVPR1B, COL6A3, ACTG1, TXLNA, ACVR1B, IL4, NENF, ARID5B, CXCR2, IL17RE, DUSP9, ASXL1, RNF111, CD40LG, CTSG, DES, BMP4, AGRP, ANGPT1, RHBG, IL22, COPB2, SDC2, MC3R, TNFRSF1B, RNASE3, TMPRSS6, NR1H2, TSHR, KDM5C, PPARD, EPHA4, SLC6A3, CASP8, CTLA4, HIVEP1, NFATC1, INHA, KDM5A, CD81, MCHR2, CCL21, NEDD4, TAF4, SMARCA1, NAT10, FAM3D, KIR2DL4, AQP9, NFKBIZ, IL17RA, CLDN4, BCL3, INHBC, NDP, UTS2B, CKLF, ARHGAP5, FGF2, NTF3, CST4, DEFA3, NCAPG2, CXCL8, HNF4A, RB1, SCG2, CD28, TMEM199, DDX5, ATG7, GIPR, CBLC, CCND2, EDEM1, TPTE, PDZD2, PIK3C2B, TTC14, ADRB2, AHSA1, LUC7L2, CALCA, CSH2, FGF4, FAM50A, HAX1, MTCP1, FGF12, HAVCR2, TNFSF8, HLA-DQB1, EDNRB, RCL1, GRM8, S1PR1, HERC1, KCNH2, CHD1, AZGP1, RNASE7, PLXNA3, IFNGR2, SAMD9L, NFATC2, NCR2, CHD5, FLT4, SYTL1, AKT3, CNTF, FAT1, PLAUR, PTN, MCHR1, ADORA2A, CMTM2, PVRIG, ECD, CREBBP, PTHLH, IFNGR1, SEMA4C, TSHB, FCN2, SST, TRPS1, IL26, BTC, MYT1L, NOX5, ARID1B, IKBKG, TLR3, CUL3, KLKB1, ESRRB, GDF10, GALP, AXIN1, PROK2, APOBEC3F, EDN1, ADIPOR1, GIP, SCAF4, PDSS2, IGF2, TRIM27, CSRP1, EGFR, TXK, IL23A, FAAH2, TG, PIK3CG, SMAD4, CCND1, HSPA6, CCR4, PLXNB1, CD276, ZNF217, BAX, MSR1, WNK1, HLA-DPA1, NPY, TFAP2A, CALR, KLRC3, OSGIN1, GPHA2, KIT, TNFRSF11B, SEMA3E, AVPR1A, PF4V1, MANF, SPP1, PCSK1, KIR3DL1, PTGER2, SEMA6D, FIGNL1, GFRAL, FGR, MAP4K3, EED, BTN2A1, TRIM5, CCNE1, GHRL, CD247, PPARG, FGF13, CD80, ATP4A, IFNA21, LTA, LIMS1, ZFHX4, NGF, RB1CC1, VIPR2, HMCN1, GDF3, SORT1, PDCD1, NFKB1, AKT1, MX2, GUSB, CCL3L3, PHKA2, IL18RAP, MEN1, SEMA6C, BCOR, LGALS9, VIM, HTR3B, RNF213, EPPIN, CCL27, GNAS, LBP, NR2E1, CHUK, DCAF4L2, GNLY, HLA-G, IL18, IL7, INSL5, NFKBIB, IL31, ENDOU, TLR7, ANGPTL7, MAGEA6, NRG4, NRAS, IL1B, TPO, FOXA1, CXCR3, TNFRSF10A, PTPN6, PTH1R, CSF3, AJUBA, FGFR3, PTH2R, CTSS, ZFP36L2, BCL10, ISG20, SLITRK3, LYN, VTN, ANGPTL5, ZBTB7B, FAS, FGF6, SEMA4D, PGC, CD160, LLGL2, GNRH2, PPP3CC, TGFB1, RXFP1, NSD1, CCR6, MASP1, COL11A1, MAP3K1, NR2F2, OSMR, MC4R, LTB4R, TYRP1, ARRB1, NOTCH3, NR0B1, ATP13A4, SOS1, SLIT1, TNFSF14, ELF3, ADAMTS12, ISG15, RORA, IL31RA, IL13RA1, VAV1, ABCD1, VEGFB, SPOP, JAK1, PCBP2, CYBB, CGB1, EDN2, CD1E, EZH2, ACVR2A, GDF11, JAG1, RNF216, RORC, ERCC6, CD209, SEMA6A, CD8A, CHP2, PXDNL, DEFB1, EPHA2, KCNQ5, IFNL2, NRG1, GRHL2, AGTR2, IFNA4, PRTN3, FGFRL1, TBK1, SMAD2, PGR, FGF7, ARHGAP35, RBM15, ADAR, HBEGF, CREB1, PRDX1, PRPF4B, NEO1, IL36G, PDGFRA, RABEP2, COL4A1, CDYL, ACVRL1, FBXW7, ILK, GCG, ANXA6, LLGL1, AEN, CSH1, SYK, PLXNB3, INSL6, GH1, LALBA, WWP1, CSF1R, BCORL1, BACH2, HCN1, CLCN4, KDR, CALCR, INHBB, CXCR6, ADM, TDGF1, CCL16, VTCN1, RNF103, NTS, IL22RA1, THSD7A, NR3C1, ATRX, CACYBP, NR1D1, PTGER4, PDGFRB, BRCA1, KLF5, IL11, ARHGEF18, AVP, IDO1, IL25, CAMP, ITGAV, SDC1, KAT6A, PDCD1LG2, RADIL, SOCS3, ESR2, IL10, PLXNB2, TBX3, ANGPTL3, SYNE1, TCHH, ATXN3L, RPGR, PDGFD, GMFG, CALCB, MAPK8, CALCRL, RIMS2, F2R, AURKA, EREG, IL13RA2, CCL23, SLC29A3, CYSLTR1, GREM2, TMEM127, INTS12, PIK3CD, PTGER3, MLN, AKAP13, NRG2, RBM10, SNX8, AMELX, MGAT5B, PTGER1, CNOT1, GKN1, SMURF2, ALB, GDNF, NCR3, PTPRD, SRRT, IL17RC, MAP3K14, IL37, CCL1, ATR, ATP6AP1, IL2RB, MTMR8, DUOX2, ZSWIM3, TMEM208, OGFR, TANK, RELA, PLXNA2, NR1D2, JUND, HCK, CER1, CTSL, C3AR1, HGF, MAPK1, HFE, VAV2, DDX17, CLIP1, KRAS, RNF43, MAGEC2, DMD, NPR1, KDM2A, ASH1L, LEP, CCRL2, KIR2DL3, MIOS, IGF2R, BMP1, PREX2, RSAD2, CMA1, COL12A1, BECN1, NOD1, RETNLB, TCF4, HDAC1, TNFRSF10B, BTN2A2, IFNA10, USP28, PHF6, CD14, AXIN2, TNFRSF19, ZNF572, NFE2L2, CD79A, PRRX1, KCNA6, PDGFB, BRAF, RPE, ANGPT4, GPR174, FGF8, FAT4, KLRD1, FGF14, IL13, TACR1, PRLH, TNFSF18, IRF2BP2, LMBR1, AMER1, HSPA4, LEPR, ZC4H2, IL5, MLNR, PLA2G2A, TERT, GFAP, OAS1, IWS1, SPAG17, FBN2, PRKCI, CAMTA1, IL10RB, PAK1, PRPF3, SDC4, KITLG, FGA, METTL3, OPRK1, NR4A3, ITGB2, TNC, CMTM5, PTGFR, MAPK14, CGB5, TENM2, YEATS4, LACRT, NR2C2, SCN5A, SDHD, MET, MYH4, BTK, HSF1, CCR1, PRKAR1A, ERBB4, DSC1, FGF16, IL2, JUN, IL33, ANKRD16, PRDX2, SKP2, PTK2B, ZCCHC24, USP13, C5AR2, BTBD11, IL1R1, CYLD, RAC1, IL27, KEAP1, NR1H4, DKK1, BRD4, ATAD2, RPS6KA3, ESR1, RXRG, NUDT11, CXCL11, GREM1, TP53BP1, LTB4R2, TLR1, IFNA7, IFNE, SLIT2, TEX14, VHL, GLP1R, CSF1, FLT1, HLA-A, SLC10A3, PTAFR, DNMT1, NRP2, CCND3, NOX3, KLRC2, SOS2, SECTM1, IL17F, BMP2, REG1A, SNRNP48, SEMA3C, HLA-DRA, CCL25, ZNF131, SBDS, LANCL1, CXCL16, CHIT1, CD70, BTN3A1, SEMA4F, F2RL1, FGFR1, RASGRP1, NF1, IL36B, SERPINA3, MUC16, IL21, CRHR2, INHBE, NR2E3, ACKR3, INO80, FOS, ADIPOQ, SLC1A3, NCBP1, IL18R1, ATG3, CYSLTR2, MAP2K1, FCGR3B, TGFB2, IFNA5, TNFSF9, HLA-E, PTK2, IL23R, GAL, PMCH, DEFA5, HDGF, TSLP, IFNA6, NRXN2, BPIFA2, GSDMC, HTR3C, ANGPTL2, TNF, GPR32, AGT, IL5RA, SCN10A, ASXL2, FZD1, COL1A2, PROK1, COL5A1, USP7, TNFRSF8, HLA-DQA1, AZIN1, TFRC, CCL2, SSX1, APOBEC3A, IRF7, GNA13, GRB2, PDGFC, PFAS, RORB, INSL4, SLC4A2, APOH, HERC2, LTBP2, CMTM7, PTEN, SDHC, CRP, CXCL3, CD27, MED23, CRIM1, SF3B1, CXCL10, MDM2, TYROBP, TGFBR2, NR3C2, GDF15, NFKBIA, PF4, INSL3, CCL13, C2CD3, IL1RL1, PTPRK, CGB7, IDH2, MAP3K4, TRAF2, INHBA, TFR2, KIR3DL3, GGCT, ADAMTS17, MYDGF, IL36A, FAM3C, KCNT2, IL19, NGFR, NOTCH4, MORC4, KLRC1, IL1A, NLRX1, KMT2D, ZBTB7A, ORM1, IL7R, LCK, SPEN, SLURP1, GDF5, AMHR2, SMG7, PIK3R1, MECOM, TAB3, GPI, TNFSF11, PKHD1L1, ITGAL, S100A7A, CD4, RASA1, DGCR8, VCAN, FOXK1, GHSR, CCR7, IL16, NODAL, CAP2, CXCL6, PTH2, EPHA5, SEMA3F, UCN3, FPR1, GDF7, GHRHR, PCDHB8, LRRK1, TNFRSF25, ING1, SHC2, TPT1, KL, LTBR, BDNF, IFNA14, THRB, ESRRG, DHX58, AGTR1, GNAQ, C10orf54, CHGB, SRC, AR, APC, ANGPTL6, XCR1, RET, STK31, ANGPTL1, KIR2DL1, PEG3, ENG, JAG2, BTNL3, SEMA7A, CDC37L1, SOCS1, CXCL5, BRPF1, FOXR2, IFNAR2, SCTR, IKBKE, PIK3R5, LHCGR, ROBO2, IL12B, SEMA6B, GHRH, ATG14, FASLG, PTGDS, FCER1G, MED12, APLNR, LIFR, TNFRSF4, BRCC3, SEMA4A, IL17RD, CD40, PPP3CB, CCR10, CEP170, HLA-DOB, CRLF1, NCOA2, SSTR1, NCOA3, HMOX1, DOT1L, PIGT, ANKRD26, B2M, FLII, IL12RB1, APOBEC3H, FURIN, HTR3E, ZNRF3, RXFP2, CTF1, SETD2, S1PR2, ACKR1, RBM8A, IFNLR1, SMARCA4, ERCC2, SSTR2, TGFB3, ICAM1, BMP3, SEMG1, MAPK7, CORT, MAGED1, ADAMTS19, CSHL1, UNC93B1, LRTM1, NR1I3, EDNRA, LTBP4, CMTM1, TMEM132B, CDK14, PPP2R1A, PSPN, NR4A1, MSH2, AGER, NDRG1, CLCF1, DAXX, ATM, XCL2, DMBT1, HLA-DPB1, GDF6, HLA-C, PNOC, GUCA2A, IL17RB, RHOB, KMT2C, IFNA13, CEACAM1, ADM2, LRRC7, CSNK1A1, DNMT3B, EPOR, IFNL3, TGFBR3, CALD1, LGR5, BPHL, NFE2L3, PAAF1, TNFRSF18, ARID1A, PPBP, HNF1A, TXNIP, DLL4, SIN3A, WNT5A, IREB2, ABCC9, NCAM2, IL1RL2, MALT1, TNFSF15, PIK3CB, TGFA, PENK, BMPR1A, CLEC11A, CX3CR1, ZNF479, BMPR2, IL36RN, CCR5, ARHGAP4, CMKLR1, NR5A1, RNF168, KMT2B, ROBO3, CDH2, TRHR, EGF, CCR3, NCR1, CSF2, MAPK3, REL, IFNA17, LEFTY1, NR0B2, CHD9, EDN3, NOTCH1, GRP, FGF18, VAV3, NR1H3, CHP1, IL1R2, MC2R, ZBTB5, CACNA1C, BMPR1B, GNAI2, ARAF, CCK, CNOT3, ACO1, TIGIT, PDGFA, GPATCH4, STK11, CD274, CCL26, CCR8, MTOR, FSHB, MARCO, LTBP3, HRG, CSK, TBL1XR1, TAP1, BMP6, TEK, COL7A1, OSTN, PRLHR, HLA-DRB5, GIGYF2, IL2RA, FUBP1, VEGFC, NES, EPB41L3, SEMA3D, KRTAP5-11, GATA3, CCL14, CHMP2B, CDKN2A, SEMA3G, HTR1A, SEMG2, TLR8, SEMA5A, LATS2, AIMP1, TRIP12, IL6R, BAG4, EEF1D, IL1RAP, BIRC5, TNFRSF10D, MYL12A, CXCL12, TRAF3, AMBN, ETV6, CD8B, FGF21, IL6ST, NPPA, C5, IL17A, PLCG1, IL15, KNG1, SHC1, EPO, CCL4, CTNNB1, ZNF536, CDK6, SCN11A, GHR, CXCR5, TNFSF13B, IRF9, STAT3, IL2RG, AZU1, AKAP8, APOB, INS-IGF2, DENND4B, COL4A2, PLAU, WDR7, CHD4, GPHB5, EIF1AX, DUSP16, KDM6A, CD86, TNFRSF21, C5AR1, LIF, TPM2, IL17D, IKBKB, GPER1, CRLF3, LHB, RXFP3, NOS1, PDGFRL, RICTOR, CCL17, APOM, IDH1, OSR2, LMO2, PHF21A, CXCR4, CTSE, ELF5, PCED1B, IFNL1, HTR3D, TP53, DPP6, HLA-DMB, NFATC3, SOX9, STAB2, HUWE1, CCL18, RUSC2, OSM, SUZ12, SLC11A1, RANBP9, IRF5, PTGDR, HLA-DOA, CTSB, LRP1B, ZNF521, ESRRA, CD1D, HIF1A, CDH1, SSX2, DMXL2, OGN, PCSK2, F8, SDHA, SHC3, CAT, IFNA8, PFKP, MUC4, OLR1, IL34, MAP2K2, RS1, BCL9L, MMP9, EPGN, COL22A1, NRG3, FRMPD4, SACS, MAGEC1, LECT2, FGF9, IL1F10, BMP15, USH2A, IFNAR1, CGB2, ROBO1, OXTR, CCL22, CARD11, CREB3L4, NCOR1, RIT1, SSTR5, NR2F6, PLCG2, BAP1, HLA-DQA2, CANX, RNASEL, BRD8, IL6, TNFSF10, IRF1, CCL24, TNFRSF1A, NR4A2, CHGA, NF2, AREG, ACTB, MAP3K13, SSH3, ERBB2, IFNB1, PPP3CA, TAC1, BMP10, MR1, VGF, ARID2, ADIPOR2, KIR3DL2, TIE1, INPPL1, FPR2, THBS2, RINL, NCOR2, CXCL13, FGFR2, NPFF, KDM1B, CCDC77, WNK2, MAPT, CD226, ABCC4, FGF5, GLP2R, CSF2RB, TNFRSF9, TRH, FAT2, CCL15, TNFRSF11A, PAN3, SIK3, NIPBL, IL15RA, ELAVL1, IL3RA, IL9R, PIK3R2, CSF2RA, IL12RB2, CRLF2 |

**Table S6. Gene list of immune-related gene signature.**

| **Classification** | **Genes** |
| --- | --- |
| Immune checkpoint | PD-1, PD-L1, PD-L2, LAG3, CTLA4, TIM3, VTCN1 |
| T-effector and INFγ pathway | GBP1, IFI16, IFI30, IFNG, IRF1, STAT1, TAP1, TAP2, FAS, PSMB9, IL15RA, GZMA, GZMB, EOMES, CXCL10, CXCL9, CXCL11, TBX21, PRF1 |
| T cell receptor | CD27, GRAP2, LCK, PTPRCAP, CCL5, IL2RB, IKZF3, CD3G, CD74, CD3D, CD8A, CD4, TIGIT |
| Tumor microenvironment | IDO1, PTGS2, IL1B, IL18, IL6, IL12A, TNF, CD73 |
| Cytolytic activity (CYT) | GZMA, PRF1 |
| Major Histocompatibility complex (MHC) | HLA-A, HLA-B, HLA-C, TAP1, TAP2, NLRC5, PSMB9, PSMB8, B2M |
| Gene expression profile (GEP) | CCL5, CD27, CD274, CD276, CD8A, CMKLR1, CXCL9, CXCR6, HLA-DQA1, HLA-DRB1, HLA-E, IDO1, LAG3, NKG7, PDCD1LG2, PSMB10, STAT1, TIGIT |

**Table S7.** **The coefficients of signature genes by the LASSO regression.**

| **Genes** | **Gene name** | **Coef** |
| --- | --- | --- |
| RARRES3 | Phospholipase A and Acyltransferase 4 | -0.00536 |
| GBP4 | Guanylate Binding Protein 4 | -0.05482 |
| GBP2 | Guanylate Binding Protein 2 | -0.1049 |
| CLEC4A | C-type Lectin Domain Family 4 Member A | -0.02784 |
| SEMA4D | Semaphorin 4D | -0.10155 |
| ADAP2 | ArfGAP with Dual PH Domains 2 | 0.11593 |
| CCL8 | C-C Motif Chemokine Ligand 8 | -0.14553 |
| NR1H3 | Nuclear Receptor Subfamily 1 Group H Member 3 | -0.10314 |
| LCP2 | Lymphocyte Cytosolic Protein 2 | -0.02153 |
| VAMP8 | Vesicle Associated Membrane Protein 8 | 0.07884 |
| FERMT3 | FERM Domain Containing Kindlin 3 | -0.00968 |
| IFITM1 | Interferon Induced Transmembrane Protein 1 | -0.0416 |
| DDX60 | DExD/H-box Helicase 60 | -0.02127 |
| LIMD2 | LIM Domain Containing 2 | 0.10432 |
| MX1 | MX Dynamin Like GTPase 1 | 0.00014 |

**Table S8 Summary of candidate drugs information.**

| **Drug name** | **Clinical trial** | **Drug category** | **Target Gene** |
| --- | --- | --- | --- |
| Sorafenib | Phase 3 | Approved, Investigational | BRAF; RAF1; FLT4; KDR; FLT1; FLT3; PDGFRB; KIT; FGFR1; RET |
| L-685458 | Not Available | Experimental | PSEN1; PSEN2; NCSTN; APH1A; APH1B; PSENEN |
| Panobinostat | Phase 1 | Approved, Investigational | HDAC1; HDAC2; HDAC3; HDAC4; HDAC5; HDAC6; HDAC7; HDAC8; HDAC9; HDAC10; HDAC11 |
| BRD-K80183349 | Not Available | Experimental | HDAC1; HDAC2 |
| BRD-K66532283 | Not Available | Experimental | HDAC1; HDAC2 |
| Cytarabine hydrochloride | Phase 3 | Approved, Investigational | POLA1; POLB; POLD1; POLE |
| BRD-K61166597 | Not Available | Experimental | HDAC1; HDAC2 |
| Merck60 | Not Available | Experimental | HDAC1; HDAC2 |
| Belinostat | Phase 2 | Approved, Investigational | HDAC1; HDAC2; HDAC3; HDAC4; HDAC5; HDAC6; HDAC7; HDAC8; HDAC9; HDAC10; HDAC11 |
| Marinopyrrole A | Not Available | Experimental | MCL1 |
| COL-3 | Phase 1 | Investigational | MMP13 |
| Rigosertib | Phase 3 | Investigational | PIK3CA; PIK3CB; PLK1 |
| Vincristine | Phase 2 | Approved, Investigational | TUBB; TUBA4A |
| Apicidin | Not Available | Experimental | HDAC1; HDAC2; HDAC3; HDAC6; HDAC8 |
| Sotrastaurin | Phase 2 | Investigational | PRKCA; PRKCB; PRKCD; PRKCE; PRKCH; PRKCQ |
| Colchicine | Phase 4 (Gout; Acute Coronary Syndrome (ACS); Coronavirus Disease 2019 (COVID-19)) | Approved | TUBB |

**Supplementary Methods**

Data Collection

We first collected multi-omics data of 450 melanoma cancer patients from the GDC TCGA data portal (https://portal.gdc.cancer.gov/), including somatic mutations (whole-exome sequencing), gene expression (RNA-seq), as well as corresponding clinical data (sex, age, survival information, etc.). The patients without sequencing and survival data were excluded from further analysis. The gene expression data of TCGA cancer patients was FPKM-normalized and subjected to log2(value+1) transformation. We then downloaded 4,872 immunologic signature gene sets (IS) from the Molecular Signatures Database (MsigDB) database (<https://www.gsea-msigdb.org/gsea/msigdb/>) ^1^ and collected driver genes related to the tumor immune microenvironment (TIME) from the Network of Cancer Genes (NCG) database (http://network-cancer-genes.org/) ^2^. To validate the association between the novel signature and the clinical benefit of ICBs, we used seven skin cutaneous melanoma (SKCM) cohorts (GSE115821 ^3^, GSE100797 ^4^, the Gide et al ^5^ (BioProject accession number PRJEB23709), GSE168204 ^6^, GSE35640 ^7^, GSE186143 ^8^ and the Nathanson et al ^9^) and one non-small-cell lung cancer (NSCLC) (the Cho et al ^10^) of patients treated with ICBs were used to further validate the association between the novel signature and the clinical benefit of ICBs. The tumor immunotherapy response was defined by the Response Evaluation Criteria in Solid Tumours 1.1 (RECIST 1.1), where patients with complete response (CR) or partial response (PR) were classified as responders; and those with stable disease (SD) or progressive disease (PD) were classified as non-responders. Especially for the GSE186143 cohort, patients were classified as a durable clinical benefit (DCB) (complete response [CR]/partial response [PR] or stable disease [SD] that lasted > 6 months) or no durable benefit (NDB) (progression of the disease [PD] or SD that lasted ≤ 6 months). Detailed information regarding the above cohorts is shown in Table S1.

Independent component analysis

We used the ICA implementation flow (Fig. 1A) described in a previous study ^11^. Briefly, we ran the scikit-learn ^12^ realization of the FastICA algorithm ^13^ 100 times using random seeds with a convergence tolerance of 10^−7^. The number of components in each iteration was set as the number of components with a 99% variance of the reconstruction calculated from the principal component analysis. The resulting source components (S) from all runs were clustered using the scikit-learn implementation of the DBSCAN algorithm ^14^ which does not require a predetermined number of clusters. In the DBSCAN analysis, we used the parameters as follows: the DISTANCE parameter (similar to the epsilon parameter in the traditional DBSCAN application) was set to the default value of 0.1 and was used to determine the maximum distance between two points that were considered to be clustered in the same cluster. Meanwhile, the MIN_FRAC parameter was maintained at the default setting of 0.5, which specifies the minimum total number of data points required to form valid clusters. This means that in our analysis, each cluster contains at least 50% of the total number of data points in the dataset. The final independent components (IC) were defined as the centroid of each cluster in S, and the weightings were defined as the centroid of their corresponding weighting vectors in W. To ensure that the final components were consistent across multiple runs, we computed the clustered components 100 times and selected the components that were identified in each run. Let us denote $\boldsymbol{E}_{\boldsymbol{nm}}$ the expression matrix of n genes measured in m samples. ICA decomposed such a matrix into a product of k statistically independent component $\boldsymbol{S}_{\boldsymbol{nk}}$ (addressed as the matrix of source component) and a weight matrix $\boldsymbol{W}_{\boldsymbol{km}}$ (matrix of samples):

$\boldsymbol{E}_{\boldsymbol{nm}}=\boldsymbol{S}_{\boldsymbol{nk}}\boldsymbol{\times}\boldsymbol{W}_{\boldsymbol{km}}$ **(1)**

Selection of immunologically relevant signature independent components

To identify the signature independent components associated with immune, we used a network-based computational method (Figure 1B). The details of network construction and ranking of components is carried out as follows.

Step (1): Construction of an IC/IS bipartite graph. To contrast our component crosstalk network, we first construct a bipartite network, where the two sets of nodes represent immune signature gene sets (IS) and independent components (IC), respectively. We considered two ICs to be functionally similar and both immunologically relevant if some of the genes in the two ICs shared at least one of the common IS. To do this, we first construct the bipartite network by defining an edge between each pair of IS and IC if the intersection of IS and IC is nonempty. We used the Jaccard index of IS and IC as the weights of the edges in this network to reflect the relationship between IS and IC, we define this weight as follows:

$\boldsymbol{J}=\frac{\left| \boldsymbol{IS}\cap\boldsymbol{IC} \right|}{\left| \boldsymbol{IS}\cup\boldsymbol{IC} \right|}$ **(2)**

Step (2): Converting the bipartite graph to a component crosstalk network. The bipartite graph constructed in step (1) can be represented algebraically in the form of an incidence matrix $\boldsymbol{J} = \left[ \boldsymbol{J}_{\boldsymbol{x},\boldsymbol{y}} \right]$, where the rows of the matrix correspond to IC and the columns respond to IS. In the bipartite network, two ICs share more neighbor IS nodes; they tend to participate in similar biological functions and implement crosstalk with each other. To further establish the component crosstalk network, we define a weighted adjacency matrix:

$\boldsymbol{A}=\boldsymbol{J}\cdot\boldsymbol{J}^{\boldsymbol{T}}$ **(3)**

Letting $\boldsymbol{N}_{\boldsymbol{IS}}$ denote the total number of IS, we see that each edge weight $\boldsymbol{A}_{\boldsymbol{x}\boldsymbol{x}^{'}}$ in our network has the form

$\boldsymbol{A}_{\boldsymbol{x}\boldsymbol{x}^{'}}=\sum_{\boldsymbol{y}}^{\boldsymbol{N}_{\boldsymbol{IS}}} \boldsymbol{J}_{\boldsymbol{IC}_{\boldsymbol{x}}\boldsymbol{IS}_{\boldsymbol{y}}}\times\boldsymbol{J}_{\boldsymbol{IC}_{\boldsymbol{x}}{'\boldsymbol{IS}}_{\boldsymbol{y}}}$ **(4)**

That is, the weight of an edge between two ICs is the sum of the contributions of all immune biological processes shared between them. Two ICs are linked in this network if and only if they share at least one biological immune process. Edge weights in the independent component network will be larger for pairs of independent components $\boldsymbol{IC}_{\boldsymbol{x}}$ and $\boldsymbol{IC}_{\boldsymbol{x}^{'}}$ that relate more to immunologic signature gene sets $\boldsymbol{IS}_{\boldsymbol{y}}$, whose contributions to both independent components are large.

Step (3): Evaluation of immune-related independent components with eigenvector centrality. Steps (1) and (2) result in a network of independent components with weighted edges, such that evidence that an independent component may play an important latent role can be expected to be reinforced by the evidence of its neighbors. Thus, an independent component is more attended to be highly correlated with cancer immunity if it is linked to more neighbor nodes and the edges have larger weight. In step (3), we applied a network propagation algorithm, the random walk with restart, to calculate the eigenvector centrality score of independent components, which is a measure to determine the significance of immune-related independent components. In this algorithm, the more central an independent component node is, the more probable it is to be visited by the random walker and result in a larger eigenvector centrality score. To implement this algorithm, we defined a probability transition matrix T by row-normalizing the adjacency matrix A. The formula is as follows:

$\boldsymbol{T}_{\boldsymbol{x}\boldsymbol{x}^{'}}=\frac{\boldsymbol{A}_{\boldsymbol{x}\boldsymbol{x}^{'}}}{\sum_{\boldsymbol{x}^{'}=\mathbf{1}}^{\boldsymbol{N}_{\boldsymbol{IC}}} \boldsymbol{A}_{\boldsymbol{x}\boldsymbol{x}^{'}}}$ **(5)**

where $\boldsymbol{N}_{\boldsymbol{IC}}$ is the total number of independent components in the network, and $\boldsymbol{T}_{\boldsymbol{x}\boldsymbol{x}^{'}}$ denotes the probability of transferring from $\boldsymbol{IC}_{\boldsymbol{x}}$ to $\boldsymbol{IC}_{\boldsymbol{x}^{'}}$. The formula of a random walk with a restart algorithm is as follows:

$\boldsymbol{\pi}^{\boldsymbol{t}+\mathbf{1}}=\left( \mathbf{1}-\boldsymbol{r} \right)\boldsymbol{T}\boldsymbol{\pi}^{\boldsymbol{t}}+\boldsymbol{r}\boldsymbol{\pi}^{\mathbf{0}}$ **(6)**

where $\boldsymbol{T}$ is the probability transition matrix from the row-normalized adjacency matrix of $\boldsymbol{A}$, and $\boldsymbol{r}$ is the restart probability which has been demonstrated to have only a slight effect on the results when it fluctuated between 0.1 and 0.9 ^15^. The default parameter $\boldsymbol{r}$ is set at 0.9 in this study. $\boldsymbol{\pi}^{\boldsymbol{t}}$ is the vector of nodes at time step t, and its xth element $\boldsymbol{\pi}_{\boldsymbol{x}}^{\boldsymbol{t}}$ it holds the probability of being at node x at time step t. The random walk with restart applied here is characterized as the limiting distribution resulting from a prolonged random walk i.e. the relative frequency of a random walker at each node in a weighted network, after walking for a long time. Therefore, in network analysis, initial values are less critical. The key is to employ random restarts to prevent slow progress due to strong community structures that might trap walkers. Thus, in this study the initial probability vector $\boldsymbol{\pi}^{\mathbf{0}}$ is constructed by assigning to each node with the same value and making their sum to be 1. After an infinite number of steps, the probability $\boldsymbol{\pi}^{\boldsymbol{t}}$ will converge to a unique steady state $\boldsymbol{\pi}$, whose ith element $\boldsymbol{\pi}_{\boldsymbol{x}}$ is defined as the EC score of the $\boldsymbol{IC}_{\boldsymbol{x}}$. A larger eigenvector centrality score for an IC indicates that the IC is more likely to be associated with cancer immunity.

To evaluate the statistical significance (P value) of the IC centrality score, we performed a bootstrap-based randomization procedure. The EC scores are calculated in the IC network, which is constructed based on the intersection of each pair of IC and IS. Therefore, we performed bootstrap resampling of all edges in the network and repeated the above algorithm to discover statistically significant ICs driven by immunization. we obtained a vector of random EC scores, $\boldsymbol{\pi}^{*}$. This process was repeated 10000 times, which obtained a set of random EC score vectors {$\boldsymbol{\pi}^{*\mathbf{1}}, \cdot\cdot\cdot,\boldsymbol{\pi}^{*\mathbf{10000}}$}. The EC scores for the original data indicated as $\boldsymbol{\pi}$ are compared with the set of random EC score vectors. We computed a P-value for each IC $\boldsymbol{IC}_{\boldsymbol{x}}$ as follows:

$\boldsymbol{P}-\boldsymbol{value}\left( \boldsymbol{IC}_{\boldsymbol{x}} \right)=\frac{\sum_{\boldsymbol{k}=\mathbf{1}}^{\mathbf{10000}} \boldsymbol{I}\left\{ {\boldsymbol{\pi}_{\boldsymbol{x}}}^{*\boldsymbol{k}}\geq\boldsymbol{\pi}_{\boldsymbol{x}} \right\}}{\mathbf{10000}}$ **(7)**

where $\boldsymbol{I}$ is the indicator function. The ICs with a P value < 0.05 are deemed as signature independent components associated with cancer immune system.

Functional annotations of independent components

We selected the genes associated with an independent component (“most contributing genes”) by thresholding the absolute projections of expression at 3 standard deviations from the mean ^16^. Independent components were assigned curated annotation by matching top z-weighted genes to functional biological information from GSEA and Enrichr. The pathway database from Enrichr utilized "WikiPathway_2023_Human", "WikiPathway_2021_Human", "WikiPathways_2019_Human", "BioPlanet_2019", "GO_Biological_Process_2023", "GO_Molecular_Function_2023", "GO_Cellular_Component_2023", and “Reactome_2022". GSEA also performed using the z-weight rank of all genes. ClusterProfiler is a universal enrichment analysis tool, that supports GO, KEGG, and gene set enrichment analysis (GSEA), and can easily visualize the enrichment analysis results. The R package “clusterProfiler” ^17^ was used to perform Gene Ontology (GO) and Kyoto Encyclopedia of Genes and Genomes (KEGG) analyses.

Calculation of TIME-driver independent components profile by integrating signature components and somatic mutation data

For somatic mutation data in melanoma patients, we converted MAF format data into a binary mutation matrix, in which each column represents a sample, and each row represents a mutated TIME-driver gene. If a TIME-driver gene mutation occurred in one sample, the element was 1; otherwise, it was 0. We then construct a TIME-driver gene mutation matrix (Figure 1C). In this study, we only extract the non-silent somatic mutations in the genomic coding regions, including missense mutations, nonsense mutations, insertions, deletions, and splice mutations. Considering that mutations in multiple genes may occur simultaneously in cancer samples and affect normal biological functions, we then defined a novel concept “TIME-driver independent components (iDICs)” to reflect the extent to which mutated genes influence biological functions by regulating gene expression. If a sample's TIME-driver gene mutation information is available, the iDICs are defined by the non-zero weights at the rows of the TIME-driver gene across ICs of $\boldsymbol{S}_{\boldsymbol{nk}}$. The TIME-driver gene in each iDIC is determined by the non-zero elements in each corresponding column of $\boldsymbol{S}_{\boldsymbol{nk}}$. For each iDIC, the total of the non-zero weights is used to evaluate the contribution of the mutation TIME-driver gene to the ICs. In $\boldsymbol{W}_{\boldsymbol{km}}$, the score corresponding to the row of the IC and the column of the sample shows the effect of the IC on the sample. We defined the impact of the mutational TIME-driver gene on the IC as $\emptyset_{\boldsymbol{i}}$:

$\emptyset_{\boldsymbol{i}}=\left\{ \begin{aligned} \boldsymbol{R}_{\boldsymbol{d}}\times\boldsymbol{W}_{\boldsymbol{id}}, \boldsymbol{d}\in\boldsymbol{D}_{\boldsymbol{i}} \\ \mathbf{0}, \boldsymbol{d}\notin\boldsymbol{D}_{\boldsymbol{i}} \end{aligned} \right.$ **(8)**

We denote the ICs driver by TIME-driver genes of sample i as set $\boldsymbol{D}_{\boldsymbol{i}}$ (i = 1, 2, 3, …, k). For each iDIC $\boldsymbol{d} \in\boldsymbol{D}_{\boldsymbol{i}}$, we define the product between $\boldsymbol{R}_{\boldsymbol{d}}$ and the weight score $\boldsymbol{W}_{\boldsymbol{id}}$ as the overall effect of the IC imposed on sample i, where $\boldsymbol{R}_{\boldsymbol{d}}=\sum_{\boldsymbol{j}=\mathbf{1}}^{\boldsymbol{n}} \boldsymbol{S}_{\boldsymbol{jd}}$.

Considering the impact of the IC itself on the disease, we introduce a variable parameter $\boldsymbol{\lambda} \left[ \mathbf{0},\mathbf{1} \right]$, where $\boldsymbol{\lambda}$ indicates the extent to which the patient is affected by the iDIC, and a larger $\boldsymbol{\lambda}$ indicates that the IC driven by the TIME-driver gene affects the patient to a greater extent, in this study the $\boldsymbol{\lambda}$ was set at 0.7. We then obtain a new profile $\boldsymbol{iDIC}_{\boldsymbol{mk}}$ representing the IC contributions to the sample weighted by TIME-driver gene expression and mutation data. The row of $\boldsymbol{iDIC}_{\boldsymbol{mk}}$ is melanoma samples and the column of $\boldsymbol{iDIC}_{\boldsymbol{mk}}$ is iDICs.

$\boldsymbol{iDIC}_{\boldsymbol{mk}}=\boldsymbol{\lambda}*\emptyset+(\mathbf{1}-\boldsymbol{\lambda})*\boldsymbol{W}$ **(9)**

Identification of iDIC subgroups

To explore novel iDIC subgroups to character the TIME of melanoma cancer patients based on iDIC, an unsupervised clustering, based on the hierarchical clustering machine learning algorithm was implemented (Figure 1D). The unsupervised clustering “Pam” method based on “Pearson” and “Ward.D” linkage was used in this analysis, executed by using the “ConsensusClusterPlus” R package ^18^, and repeated 1,000 times to ensure classification stability. The optimal number of clusters was determined by the proportion of the ambiguous clustering algorithm and the consensus heatmap ^19^, in this study we grouped melanoma patients into clusterA and clusterB groups. The different expression genes (DEGs) between different iDIC subgroups were identified using the “limma” R package ^20^. The genes with a Benjamini-Hochberg false discovery rate adjusted p-value < 0.05 and absolute fold-change > 1 were identified as the significant differential genes between two iDIC subgroups. The Boruta algorithm was used to further select important genes for the iDIC subgroups ^21^. Survival analysis for the iDIC subgroups was performed by Kaplan-Meier curves, and their significance was assessed by the log-rank method.

We compared the differences in immune-related signature features, including immune score, cytolytic activity (CYT) score ^22^, major histocompatibility complex (MHC)-I score ^23^, and T-cell-inflamed immune gene expression profile (GEP) score ^24^, between clusterA and clusterB patients. The immune score was estimated by the “ESTIMATE” R package ^25^. The CYT score and MHC-I score were calculated by taking the mean expression of their signature genes. The GEP score was estimated by performing ssGSEA using the GEP gene list which is listed in Supplementary Table S6. To further explore the difference in immune infiltration the abundance of tumor infiltration immune cells was calculated to investigate the relationship between the TIME subgroups and cancer immunity by three algorithms including MCP-counter, CIBERSORT, and xCell in the “IOBR” R package ^26^.

Development of iDIC-based risk score signature

To further explore whether iDIC could predict the efficacy of immunotherapy, we developed a risk score signature based on the iDIC subgroups (Figure 1D). The least absolute shrinkage and selection operator (LASSO) regression was then used to further narrow the scope of the important genes of iDIC subgroups obtained by Boruta algorithm by selecting the genes with optimal prediction, and 10-fold cross-validation was used to determine the regression shrinkage parameter. The iDIC-based classification, a risk model termed iDIC-based scoring system (iDICss), was calculated by the formula:$\boldsymbol{iDICss}=\sum\boldsymbol{\beta}_{\boldsymbol{i}}{\times\boldsymbol{G}}_{\boldsymbol{i}} (\mathbf{10})$, where the $\boldsymbol{\beta}_{\boldsymbol{i}}$ is the coefficient of gene i in the LASSO regression and the $\boldsymbol{G}_{\boldsymbol{i}}$ represents the expression of gene i. Then the median value of the iDICss was used to stratify patients into high-risk and low-risk groups. Kaplan-Meier survival analysis was used to estimate two subgroups’ survival distributions and the performance of the iDICss was investigated by the ROC curve. The Mann-Whitney U test was further performed to test the difference in objective response rate (ORR) for immunotherapy between high-risk and low-risk groups.

**Assessment of drug sensitivity and efficacy of iDICss in melanoma**

To further explore the actionability of iDICss signature for clinical applications, we seek to screen out potential therapeutic agents highly correlated with iDICss, which may have therapeutic implications for high-risk SKCM patients. For this purpose, we collected expression data of cell lines and corresponding drug IC50 values from the Cancer Cell Line Encyclopedia (CCLE, https://sites.broadinstitute.org/ccle/), CellMiner (https://discover.nci.nih.gov/cellminer/home.do) and Cancer Therapeutics Response Portal (CTRP, https://portals.broadinstitute.org/ctrp/) databases, respectively. Then, we calculated iDICss for each cell line based on expression data and evaluated the correlation between iDICss and drug sensitivity (IC50 values). We selected drugs with a significant negative correlation between sensitivity and iDICss as candidate drugs, with adjust p-values less than 0.05 calculated by Spearman correlation.

**Comparison with existing models in inferring prognosis and immunotherapeutic outcomes**

To highlight the superior performance of iDICss in predicting prognosis and immunotherapeutic outcomes in melanoma patients, we conducted comparisons between iDICss and 117 combined machine learning models as well as published transcriptomic features. Moreover, we compare the efficacy of iDICss with a framework for unsupervised integration of multi-omics datasets (MOFA) ^27^ as a predictor of clinical outcome. Specifically, we employed univariate Cox regression to filter differentially expressed genes between clusterA and clusterB, retaining genes with a p value less than 0.01 for constructing machine learning models. We employed ten distinct machine learning algorithms, including stepwise Cox, CoxBoost, ridge regression, Random Survival Forest (RSF), Generalized Boosted Regression Models (GBMs), Survival Support Vector Machines (Survival-SVMs), LASSO, Supervised Principal Components (SuperPC), Partial Least Squares Cox regression (plsRcox), and Elastic Net (Enet). The procedure for model integration was as follows: first, prognostic genes in the TCGA-SKCM cohort were identified using univariate Cox regression; then, 117 machine learning combinations were performed on prognostic genes in the TCGA SKCM cohort in order to fit the predictive models based on the Leave-One-Out Cross-Validation (LOOCV) framework; subsequently, all models were tested in three validation datasets (GSE115821, GSE10797 and Gide cohort); finally, Harrell's consistency index (C-index) was calculated for each model across all validation datasets, and the model with the highest average C-index was considered to be the best model.

We then evaluated the predictive performance of the iDICss in the immunotherapy cohort. To this end, we further compared the iDICss against those of previously reported transcriptome-based predictive signatures, including PD-1 ^28^, PD-L1 ^29^, plasma.cells (plasma) ^30^, immuno-predictive score (IMPRES) ^3^, TCF7 ^31^ , innate anti-PD-1 resistance (IPRES) ^32^, epithelial-to-mesenchymal transition (EMT) ^33^, anti-CTLA4 resistance MAGE gene (CRAM) ^34^, LRRC15+ carcinoma-associated fibroblasts (CAF) ^35^ and immune-related genes (IRGs) ^36^.

**Supplementary References**

1. Subramanian A, Tamayo P, Mootha VK, et al. Gene set enrichment analysis: a knowledge-based approach for interpreting genome-wide expression profiles. *Proc Natl Acad Sci U S A.* 2005;102(43):15545-15550.

2. Repana D, Nulsen J, Dressler L, et al. The Network of Cancer Genes (NCG): a comprehensive catalogue of known and candidate cancer genes from cancer sequencing screens. *Genome Biol.* 2019;20(1):1.

3. Auslander N, Zhang G, Lee JS, et al. Robust prediction of response to immune checkpoint blockade therapy in metastatic melanoma. *Nat Med.* 2018;24(10):1545-1549.

4. Lauss M, Donia M, Harbst K, et al. Mutational and putative neoantigen load predict clinical benefit of adoptive T cell therapy in melanoma. *Nat Commun.* 2017;8(1):1738.

5. Gide TN, Quek C, Menzies AM, et al. Distinct Immune Cell Populations Define Response to Anti-PD-1 Monotherapy and Anti-PD-1/Anti-CTLA-4 Combined Therapy. *Cancer Cell.* 2019;35(2):238-255 e236.

6. Du K, Wei S, Wei Z, et al. Pathway signatures derived from on-treatment tumor specimens predict response to anti-PD1 blockade in metastatic melanoma. *Nat Commun.* 2021;12(1):6023.

7. Ulloa-Montoya F, Louahed J, Dizier B, et al. Predictive gene signature in MAGE-A3 antigen-specific cancer immunotherapy. *J Clin Oncol.* 2013;31(19):2388-2395.

8. Lozano AX, Chaudhuri AA, Nene A, et al. T cell characteristics associated with toxicity to immune checkpoint blockade in patients with melanoma. *Nat Med.* 2022;28(2):353-362.

9. Kong J, Ha D, Lee J, et al. Network-based machine learning approach to predict immunotherapy response in cancer patients. *Nat Commun.* 2022;13(1):3703.

10. Cho JW, Hong MH, Ha SJ, et al. Genome-wide identification of differentially methylated promoters and enhancers associated with response to anti-PD-1 therapy in non-small cell lung cancer. *Exp Mol Med.* 2020;52(9):1550-1563.

11. McConn JL, Lamoureux CR, Poudel S, Palsson BO, Sastry AV. Optimal dimensionality selection for independent component analysis of transcriptomic data. *BMC Bioinformatics.* 2021;22(1):584.

12. Tanaka T. [[Fundamentals] 5. Python+scikit-learn for Machine Learning in Medical Imaging]. *Nihon Hoshasen Gijutsu Gakkai Zasshi.* 2023;79(10):1189-1193.

13. Hyvarinen A. Fast and robust fixed-point algorithms for independent component analysis. *IEEE Trans Neural Netw.* 1999;10(3):626-634.

14. Ester M, Kriegel H-P, Sander J, Xu X. A density-based algorithm for discovering clusters in large spatial databases with noise. Proceedings of the Second International Conference on Knowledge Discovery and Data Mining; 1996; Portland, Oregon.

15. Kohler S, Bauer S, Horn D, Robinson PN. Walking the interactome for prioritization of candidate disease genes. *Am J Hum Genet.* 2008;82(4):949-958.

16. Teschendorff AE, Journee M, Absil PA, Sepulchre R, Caldas C. Elucidating the altered transcriptional programs in breast cancer using independent component analysis. *PLoS Comput Biol.* 2007;3(8):e161.

17. Wu T, Hu E, Xu S, et al. clusterProfiler 4.0: A universal enrichment tool for interpreting omics data. *Innovation (Camb).* 2021;2(3):100141.

18. Wilkerson MD, Hayes DN. ConsensusClusterPlus: a class discovery tool with confidence assessments and item tracking. *Bioinformatics.* 2010;26(12):1572-1573.

19. Senbabaoglu Y, Michailidis G, Li JZ. Critical limitations of consensus clustering in class discovery. *Sci Rep.* 2014;4:6207.

20. Ritchie ME, Phipson B, Wu D, et al. limma powers differential expression analyses for RNA-sequencing and microarray studies. *Nucleic Acids Res.* 2015;43(7):e47.

21. Kursa MB, Rudnicki WR. Feature Selection with the Boruta Package. *Journal of Statistical Software.* 2010;36(11):1 - 13.

22. Rooney MS, Shukla SA, Wu CJ, Getz G, Hacohen N. Molecular and genetic properties of tumors associated with local immune cytolytic activity. *Cell.* 2015;160(1-2):48-61.

23. Liu D, Schilling B, Liu D, et al. Integrative molecular and clinical modeling of clinical outcomes to PD1 blockade in patients with metastatic melanoma. *Nat Med.* 2019;25(12):1916-1927.

24. Cristescu R, Mogg R, Ayers M, et al. Pan-tumor genomic biomarkers for PD-1 checkpoint blockade-based immunotherapy. *Science.* 2018;362(6411).

25. Aran D, Sirota M, Butte AJ. Systematic pan-cancer analysis of tumour purity. *Nat Commun.* 2015;6:8971.

26. Zeng D, Ye Z, Shen R, et al. IOBR: Multi-Omics Immuno-Oncology Biological Research to Decode Tumor Microenvironment and Signatures. *Front Immunol.* 2021;12:687975.

27. Argelaguet R, Velten B, Arnol D, et al. Multi-Omics Factor Analysis-a framework for unsupervised integration of multi-omics data sets. *Mol Syst Biol.* 2018;14(6):e8124.

28. Kleffel S, Posch C, Barthel SR, et al. Melanoma Cell-Intrinsic PD-1 Receptor Functions Promote Tumor Growth. *Cell.* 2015;162(6):1242-1256.

29. Chen G, Huang AC, Zhang W, et al. Exosomal PD-L1 contributes to immunosuppression and is associated with anti-PD-1 response. *Nature.* 2018;560(7718):382-386.

30. Patil NS, Nabet BY, Muller S, et al. Intratumoral plasma cells predict outcomes to PD-L1 blockade in non-small cell lung cancer. *Cancer Cell.* 2022;40(3):289-300 e284.

31. Jia D, Wang Q, Qi Y, et al. Microbial metabolite enhances immunotherapy efficacy by modulating T cell stemness in pan-cancer. *Cell.* 2024;187(7):1651-1665 e1621.

32. Hugo W, Zaretsky JM, Sun L, et al. Genomic and Transcriptomic Features of Response to Anti-PD-1 Therapy in Metastatic Melanoma. *Cell.* 2017;168(3):542.

33. Thompson JC, Hwang WT, Davis C, et al. Gene signatures of tumor inflammation and epithelial-to-mesenchymal transition (EMT) predict responses to immune checkpoint blockade in lung cancer with high accuracy. *Lung Cancer.* 2020;139:1-8.

34. Shukla SA, Bachireddy P, Schilling B, et al. Cancer-Germline Antigen Expression Discriminates Clinical Outcome to CTLA-4 Blockade. *Cell.* 2018;173(3):624-633 e628.

35. Dominguez CX, Muller S, Keerthivasan S, et al. Single-Cell RNA Sequencing Reveals Stromal Evolution into LRRC15(+) Myofibroblasts as a Determinant of Patient Response to Cancer Immunotherapy. *Cancer Discov.* 2020;10(2):232-253.

36. Yang S, Wu Y, Deng Y, et al. Identification of a prognostic immune signature for cervical cancer to predict survival and response to immune checkpoint inhibitors. *Oncoimmunology.* 2019;8(12):e1659094.
